# Antiplatelet monotherapy maintenance strategies after percutaneous coronary intervention in patients with coronary artery disease at high bleeding risk: a network meta-analysis

**DOI:** 10.3389/fcvm.2026.1941814

**Published:** 2026-10-28

**Authors:** Xiao Zhang, YuJie Wang, YuXin Guo, XiangJun Li

**Affiliations:** 1 Department of Cardiology, Huaihe Hospital of Henan University, Kaifeng, Henan, China; 2 Department of Pediatrics, Huaihe Hospital of Henan University, Kaifeng, Henan, China

**Keywords:** antiplatelet monotherapy, dual antiplatelet therapy, high bleeding risk, network meta-analysis, percutaneous coronary intervention

## Abstract

**Background:**

The optimal antiplatelet monotherapy maintenance strategy after percutaneous coronary intervention (PCI) remains uncertain in patients with coronary artery disease at high bleeding risk, particularly among those not receiving oral anticoagulant therapy. We performed a network meta-analysis to compare the safety and efficacy of different antiplatelet monotherapy strategies after an ultrashort course of dual antiplatelet therapy (DAPT).

**Methods:**

We searched PubMed, Embase, Web of Science, Cochrane CENTRAL, and ClinicalTrials from database inception to July 1, 2026. Randomized controlled trials and subgroup analyses of randomized trials were eligible if they enrolled high bleeding risk patients undergoing PCI who were not receiving oral anticoagulant therapy and compared antiplatelet monotherapy strategies with DAPT or with another monotherapy strategy. The primary safety outcome was BARC major bleeding, and the primary efficacy outcome was major adverse cardiovascular events (MACE). Secondary outcomes included myocardial infarction, all-cause death, and stroke. Odds ratios (ORs) with 95% confidence intervals (CIs) were estimated using a frequentist random-effects network meta-analysis.

**Results:**

All six included studies informed the drug-specific network meta-analysis. Compared with DAPT, the ORs for major bleeding were 0.26 (95% CI, 0.05–1.42) for aspirin, 0.28 (95% CI, 0.07–1.15) for clopidogrel, and 0.42 (95% CI, 0.17–1.08) for ticagrelor monotherapy. All three confidence intervals were wide and compatible with both lower and higher odds of major bleeding. No significant differences were observed for MACE, all-cause death, or stroke. The higher MI estimates for clopidogrel and aspirin require cautious interpretation given the sparse network and indirect evidence.

**Conclusions:**

In patients at high bleeding risk undergoing PCI who were not receiving oral anticoagulant therapy, the available evidence was insufficient to determine whether antiplatelet monotherapy after ultrashort DAPT reduces major bleeding compared with DAPT. Ticagrelor monotherapy showed numerically favorable estimates for MACE and major bleeding compared with DAPT, but neither comparison reached statistical significance. However, the sparse evidence network and low certainty of evidence warrant cautious interpretation.

**Systematic Review Registration:**

https://www.crd.york.ac.uk/prospero/display_record.php?ID=CRD420261448916, PROSPERO CRD420261448916.

## 1 Introduction

Dual antiplatelet therapy (DAPT), consisting of aspirin and a P2Y12 inhibitor, remains a cornerstone of antithrombotic treatment after percutaneous coronary intervention (PCI) (1–3). This strategy reduces recurrent ischemic events and stent thrombosis, particularly during the early period after stent implantation. However, prolonged exposure to DAPT inevitably increases bleeding risk, and bleeding events after PCI are associated with subsequent morbidity and mortality (4, 5). Therefore, optimizing the duration and intensity of antiplatelet therapy has become a central issue in contemporary PCI practice, especially in patients in whom the bleeding hazard is high (6).

Patients at high bleeding risk (HBR) represent a clinically important population in whom the balance between ischemic protection and bleeding avoidance is particularly difficult (7, 8). Although newer-generation drug-eluting stents and improved procedural techniques have made abbreviated DAPT more feasible, the optimal maintenance antiplatelet strategy after DAPT discontinuation remains uncertain (9). In clinical practice, several antiplatelet monotherapy strategies may be considered, including aspirin monotherapy and P2Y12 inhibitor monotherapy with different agents. These strategies differ in pharmacological profile, antithrombotic potency, bleeding liability, and supporting evidence. Direct head-to-head comparisons among all available monotherapy strategies in HBR patients after PCI remain limited.

Previous randomized trials have evaluated abbreviated DAPT followed by antiplatelet monotherapy in selected PCI populations, and conventional pairwise meta-analyses have generally suggested that shorter DAPT strategies may reduce bleeding without a clear excess in ischemic events (10–16). Nevertheless, these analyses do not fully address the comparative effectiveness and safety of different monotherapy maintenance options in HBR patients. In addition, patients requiring oral anticoagulation represent a distinct antithrombotic population, because concomitant anticoagulant therapy substantially alters both bleeding risk and treatment decision-making (17, 18). Evidence focused on HBR patients who do not require oral anticoagulation may therefore provide a clearer assessment of antiplatelet monotherapy strategies themselves. Moreover, endpoints such as major adverse cardiovascular events (MACE) and major bleeding are not uniformly defined across trials, which complicates direct interpretation of pooled evidence. A network meta-analysis may help integrate direct and indirect evidence and provide a structured comparison of available antiplatelet monotherapy strategies when conventional pairwise comparisons are incomplete.

In this study, we conducted a systematic review and network meta-analysis of six randomized controlled trials to compare antiplatelet monotherapy maintenance strategies after PCI in patients with coronary artery disease at high bleeding risk, excluding populations requiring oral anticoagulation. The primary efficacy outcome was MACE, and the primary safety outcome was major bleeding, prioritized as Bleeding Academic Research Consortium (BARC) type 3 or 5 bleeding when available. Secondary outcomes included all-cause death, cardiovascular death, myocardial infarction, and stroke. This analysis aimed to clarify the relative efficacy and safety profiles of available monotherapy strategies in HBR patients after PCI and to inform individualized antiplatelet treatment decisions in patients whose long-term antithrombotic management is not driven by an indication for oral anticoagulation.

## 2 Methods

### 2.1 Study design and reporting

This systematic review and network meta-analysis was conducted to compare antiplatelet monotherapy maintenance strategies after percutaneous coronary intervention (PCI) in patients with coronary artery disease at high bleeding risk (HBR). The study was performed in accordance with the Preferred Reporting Items for Systematic Reviews and Meta-Analyses extension statement for network meta-analyses (PRISMA-NMA). The protocol was [registered in PROSPERO; registration number: CRD420261448916.

### 2.2 Search strategy and study selection

A comprehensive literature search was conducted in PubMed, Embase, Web of Science, Cochrane CENTRAL, and ClinicalTrials from inception to July 1, 2026. The search strategy combined terms related to PCI, high bleeding risk, dual antiplatelet therapy, aspirin, P2Y12 inhibitors, antiplatelet monotherapy, randomized controlled trials, and abbreviated DAPT. Reference lists of eligible studies and relevant reviews were also screened to identify additional studies. The database-specific search strategies are provided in the Supplementary Material entitled “Search strategy”.

Two investigators independently screened titles and abstracts, followed by full-text review of potentially eligible articles. Disagreements were resolved by discussion or by consultation with a third investigator.

### 2.3 Eligibility criteria

Studies were eligible if they met the following criteria: (1) randomized controlled trials; (2) enrolled adult patients with coronary artery disease undergoing PCI; (3) included patients at high bleeding risk or a population meeting predefined HBR criteria; (4) compared antiplatelet monotherapy maintenance strategies after a short course of DAPT; and (5) reported at least one outcome of interest.

Studies were excluded if they were observational studies, subgroup analyses without extractable randomized comparisons, conference abstracts without sufficient outcome data, duplicate publications, or studies in which the antithrombotic strategy was primarily determined by an indication for oral anticoagulation. Trials or treatment arms requiring long-term oral anticoagulation were excluded because concomitant anticoagulant therapy substantially modifies bleeding and ischemic risks and may confound the comparative assessment of antiplatelet monotherapy strategies. Studies including mixed populations were considered only when data for patients not receiving oral anticoagulation could be extracted separately.

### 2.4 Interventions and network nodes

Eligible interventions were antiplatelet monotherapy maintenance strategies after PCI, including aspirin monotherapy and P2Y12 inhibitor monotherapy. P2Y12 inhibitor monotherapy was classified according to the specific agent when reported, such as clopidogrel, ticagrelor, or prasugrel monotherapy. When a study reported P2Y12 inhibitor monotherapy without specifying the agent or the distribution of agents, the treatment node was handled separately as unspecified or mixed P2Y12 inhibitor monotherapy and was not merged with drug-specific nodes in the primary drug-level analysis.

### 2.5 Outcomes

The primary efficacy outcome was major adverse cardiovascular events (MACE), as defined in each included trial. The primary safety outcome was major bleeding. When multiple bleeding definitions were reported, Bleeding Academic Research Consortium (BARC) type 3 or 5 bleeding was prioritized. If BARC 3 or 5 bleeding was unavailable, the closest trial-defined major bleeding endpoint was extracted and recorded according to the original definition.

Secondary outcomes included all-cause death, cardiovascular death, myocardial infarction, and stroke. Outcome definitions were extracted verbatim from each trial because composite ischemic outcomes and bleeding definitions may vary across studies.

### 2.6 Data extraction

Two investigators independently extracted study-level data using a standardized form. Extracted information included trial name, publication year, study design, eligibility criteria, HBR definition, sample size, baseline characteristics, PCI-related characteristics, DAPT duration before monotherapy, antiplatelet monotherapy strategy, follow-up duration, outcome definitions, and numbers of events and participants for each outcome. Data were extracted according to the intention-to-treat population whenever available. Any discrepancies were resolved by consensus.

### 2.7 Risk-of-bias assessment

The risk of bias of included randomized trials was assessed independently by two investigators using the Cochrane Risk of Bias 2 tool. The following domains were evaluated: bias arising from the randomization process, bias due to deviations from intended interventions, bias due to missing outcome data, bias in outcome measurement, and bias in selection of the reported result. Each study was judged as having low risk of bias, some concerns, or high risk of bias. Disagreements were resolved by discussion.

### 2.8 Statistical analysis

Network meta-analysis was performed for outcomes with sufficient data across the treatment network. For dichotomous outcomes, treatment effects were estimated as odds ratios (ORs) with 95% confidence intervals (CIs).

A frequentist random-effects consistency model was used to synthesize direct and indirect evidence. The model was fitted using restricted maximum likelihood, with a common between-study heterogeneity variance across treatment comparisons within each outcome. This allowed the true effect for a given treatment comparison to vary across studies. Treatment rankings were summarized using SUCRA values when available and were interpreted as exploratory.

Analyses were performed in Stata 18 using the network commands for network meta-analysis. A two-sided *P* value < 0.05 was considered statistically significant where applicable.

## 3 Results

### 3.1 Study selection

A total of 7,023 records were identified through database searches, including PubMed (*n* = 2,678), Web of Science (*n* = 74), Embase (*n* = 2,489), and Cochrane CENTRAL (*n* = 1,782). After removal of 948 duplicates, 6,075 records were screened by title and abstract. Of these, 5,839 records were excluded, and 233 reports were sought for full-text retrieval after excluding 3 duplicate studies. Seven reports could not be retrieved, leaving 226 full-text reports for eligibility assessment. After excluding 220 reports, 6 studies were finally included (Figure 1).

**Figure 1:**
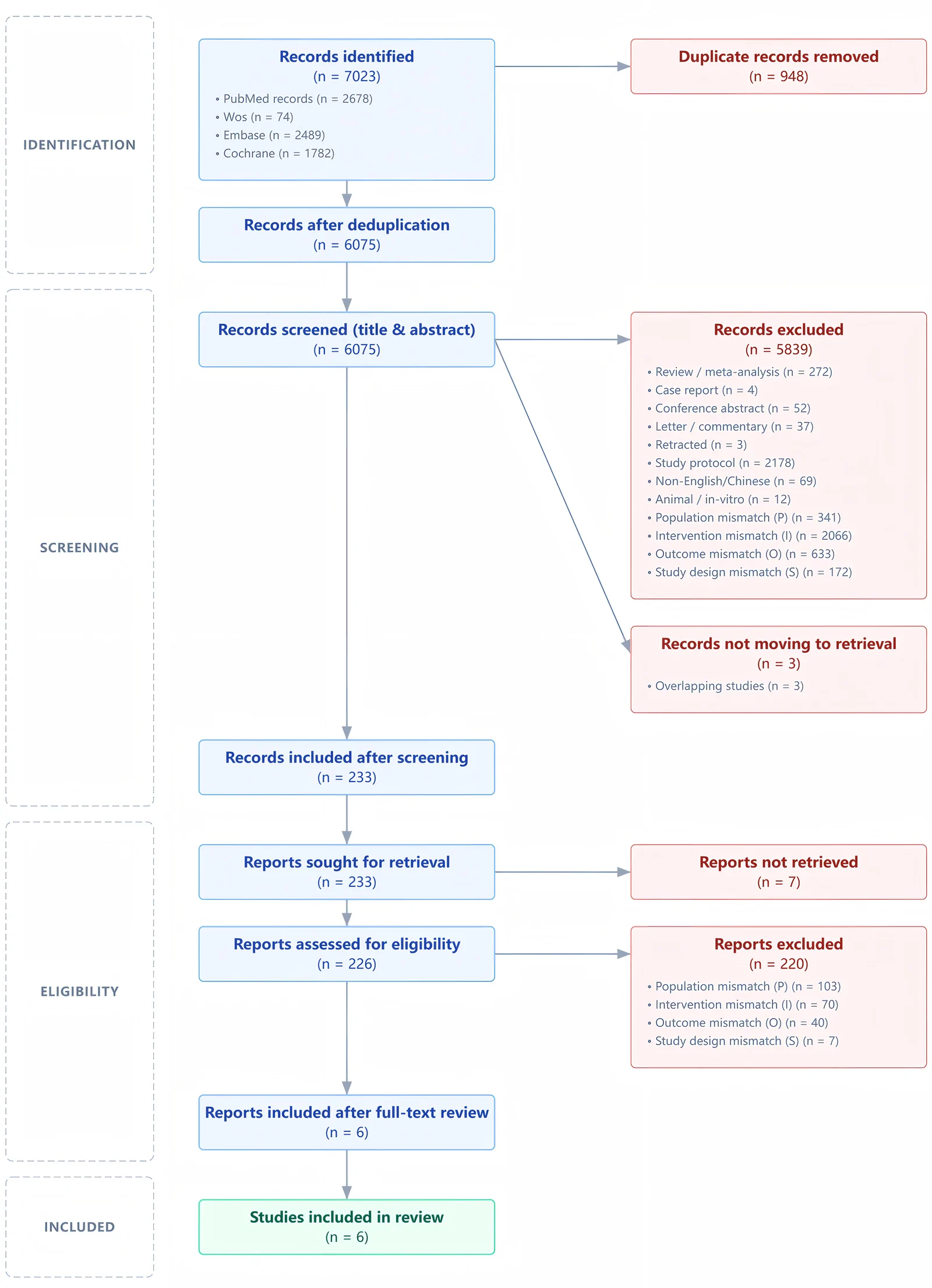
PRISMA flow diagram of the study selection process.

### 3.2 Study characteristics and network geometry

The included studies enrolled patients with coronary artery disease undergoing PCI who were at high bleeding risk and were not receiving oral anticoagulant therapy. The evidence base included original randomized controlled trials and subgroup analyses of randomized trials. All network estimates were based on 1-year follow-up data. The evaluated strategies included antiplatelet monotherapy after an ultrashort course of DAPT and standard DAPT. The basic characteristics of the six studies are shown in Table 1.

**Table 1:** Trial-specific characteristics.

Study	Year	Number of Patients	Female (%)	Mean age	Hypertension	Diabetes Mellitus	CKD
TICO	2022	453	64%	70.6 ± 9.0	69.50%	49.20%	69.30%
TWILIGHT-HBR	2021	1,064	32.10%	71.7 ± 10.7	81.40%	45.90%	59.10%
GLOBAL LEADERS	2020	838	61.80%	71.3	91.90%	100%	100%
STOPDAPT-2	2023	1,893	68.80%	76.4 ± 8.7	78.30%	38.60%	73.10%
STOPDAPT-3	2025	1,711	69.80%	77.9 ± 9.0	79.20%	45.30%	76.90%
HOST-EXAM	2024	508	56.40%	71.9 ± 9.3	77.70%	51.90%	100%
	HBR Dfinitions			Fllow-up Tme		Mnimum DAPT Dration	Source of the Sample
TICO	ARC-HBRor PRECISE-DAPT			12 months		3 month	South Korea
TWILIGHT-HBR	ARC-HBR			6 and 12month		3 month	Global
GLOBAL LEADERS	-			30 days and 3, 6, 12, 18 and 24 months		1 month	Global
STOPDAPT-2	ARC-HBR			12 months		1 month	Japan
STOPDAPT-3	ARC-HBR			12 months		1 month	Japan
HOST-EXAM	-			12and 24 months		3 month	South Korea

The drug-specific network included four treatment nodes: DAPT, aspirin monotherapy, clopidogrel monotherapy, and ticagrelor monotherapy (Figure 2). The network was connected but sparse and did not contain closed loops. The absence of closed loops precluded formal inconsistency testing. Between-study heterogeneity was estimated in the random-effects model, but the small number of studies limited the precision of heterogeneity estimation.

**Figure 2:**
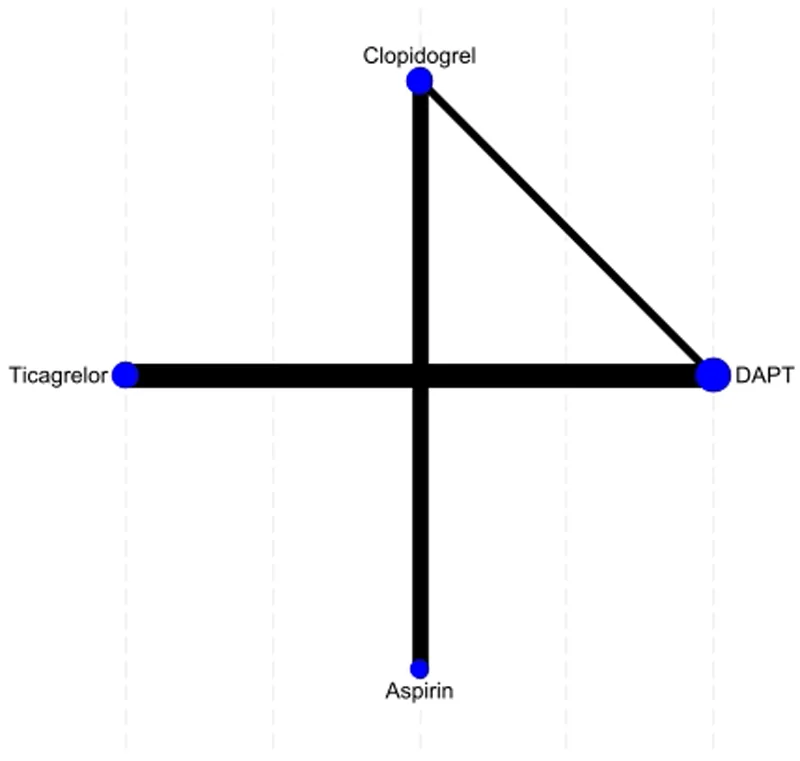
Network geometry of the drug-specific network meta-analysis.

### 3.3 Risk of bias

Risk of bias was assessed for six studies contributing to the drug-specific network. Overall, 37 of 42 domain-level judgments were rated as low risk of bias, and 5 were rated as high risk of bias (Figures 3, 4). No domain was judged as unclear risk. High-risk judgments were concentrated in the domain of blinding of participants and personnel, reflecting the open-label nature of several antiplatelet strategy trials. Other domains, including random sequence generation, allocation concealment, blinding of outcome assessment, incomplete outcome data, selective reporting, and other bias, were generally rated as low risk.

**Figure 3:**
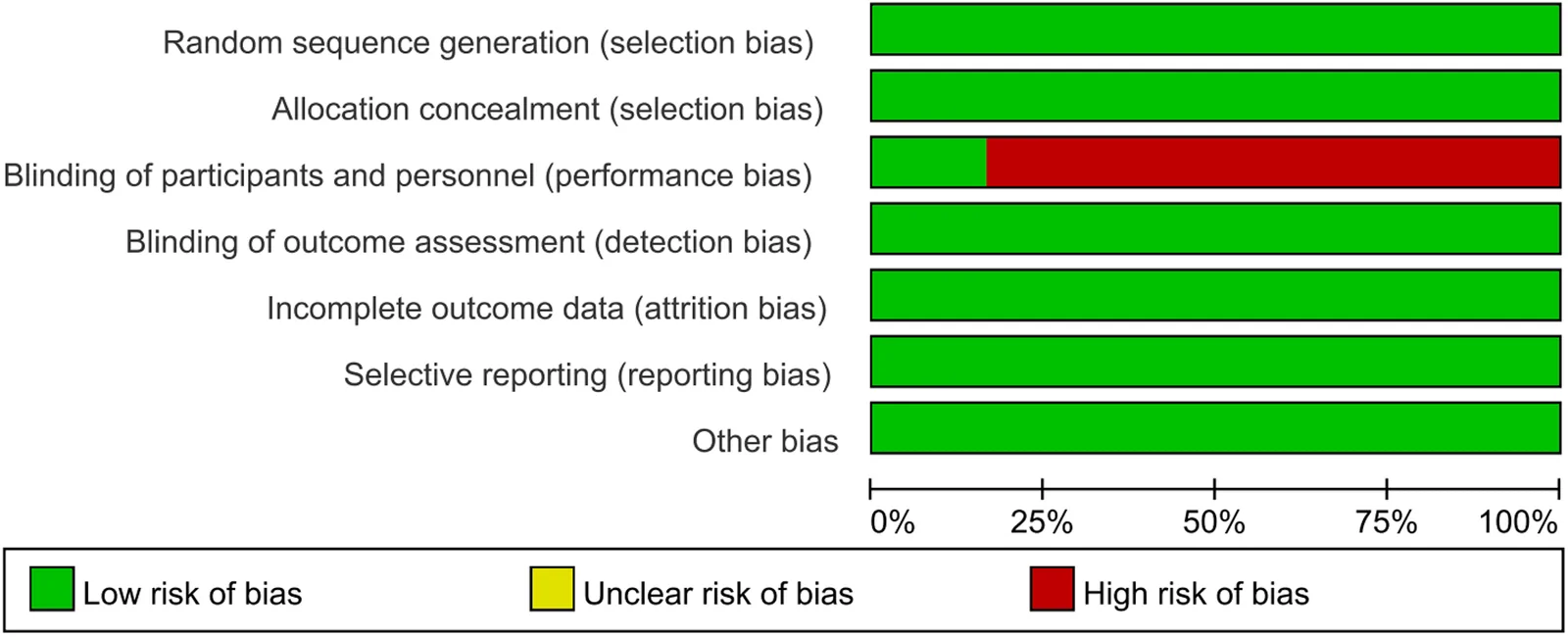
Risk of bias assessment using the cochrane risk of bias 2 (RoB 2) tool.

**Figure 4:**
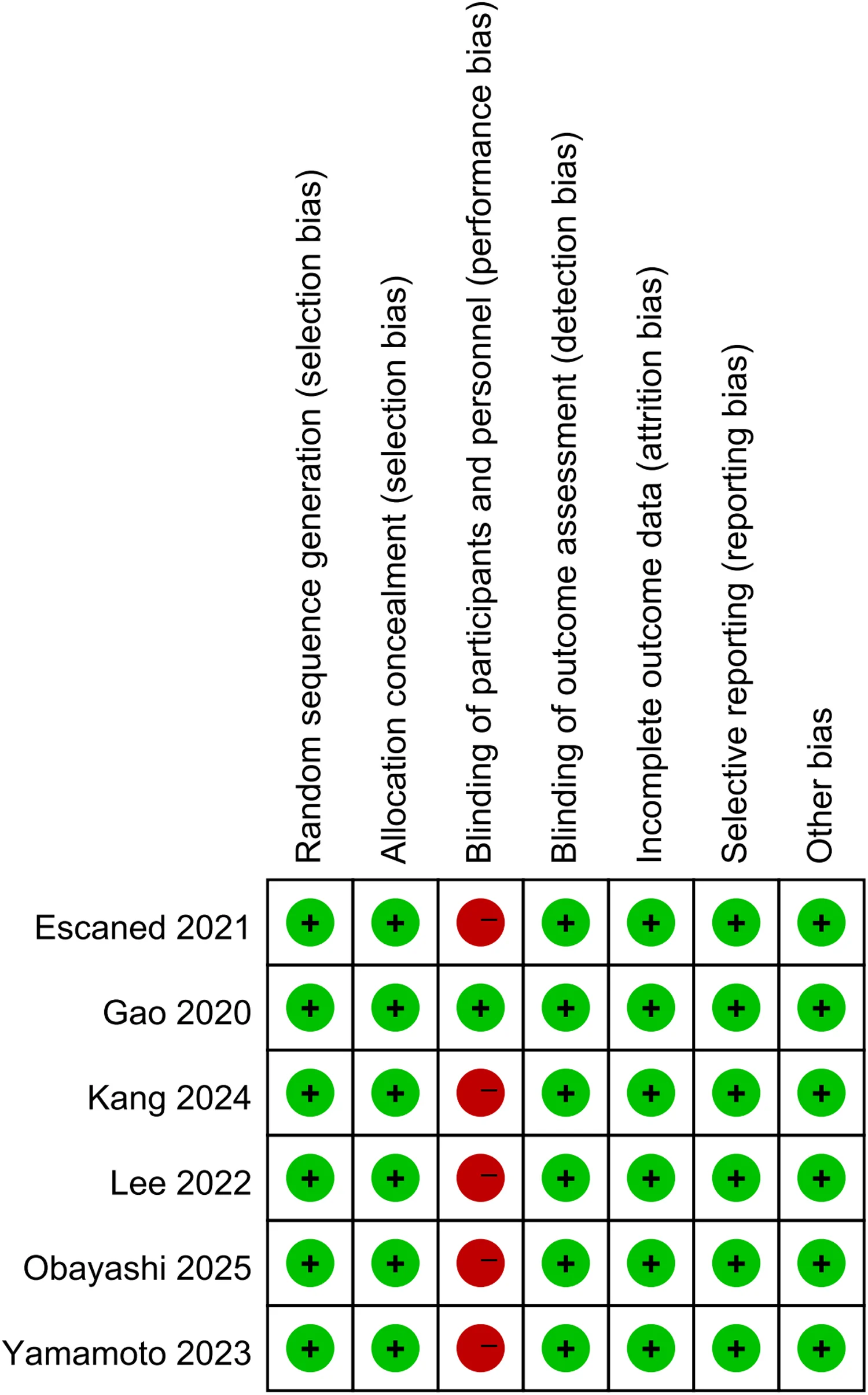
Summary of risk of bias across all included studies.

### 3.4 Major bleeding

The primary safety outcome was major bleeding, defined according to BARC criteria. Compared with DAPT, the ORs were 0.26 for aspirin monotherapy (95% CI, 0.05–1.42), 0.28 for clopidogrel monotherapy (95% CI, 0.07–1.15), and 0.42 for ticagrelor monotherapy (95% CI, 0.17–1.08) (Figure 5). All three confidence intervals were wide and included 1.

**Figure 5:**
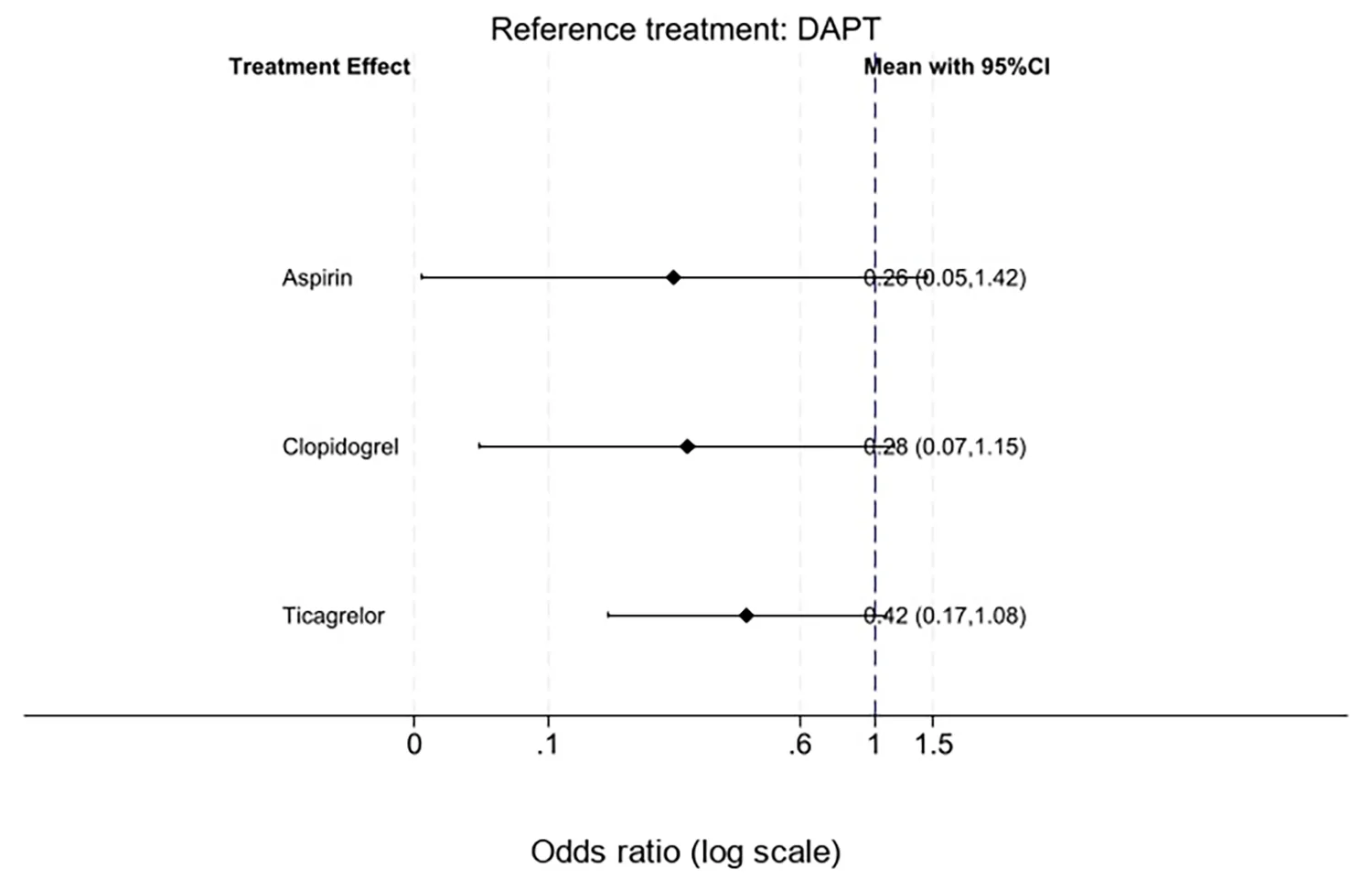
Forest plot for the primary safety outcome: major bleeding.

### 3.5 MACE

No statistically significant differences in MACE were observed between DAPT and any antiplatelet monotherapy strategy. Compared with DAPT, ticagrelor monotherapy showed a numerically lower estimate (OR, 0.83; 95% CI, 0.57–1.19), whereas clopidogrel monotherapy (OR, 1.24; 95% CI, 0.69–2.25) and aspirin monotherapy (OR, 1.33; 95% CI, 0.64–2.76) showed numerically higher estimates. All confidence intervals crossed unity (Figure 6).

**Figure 6:**
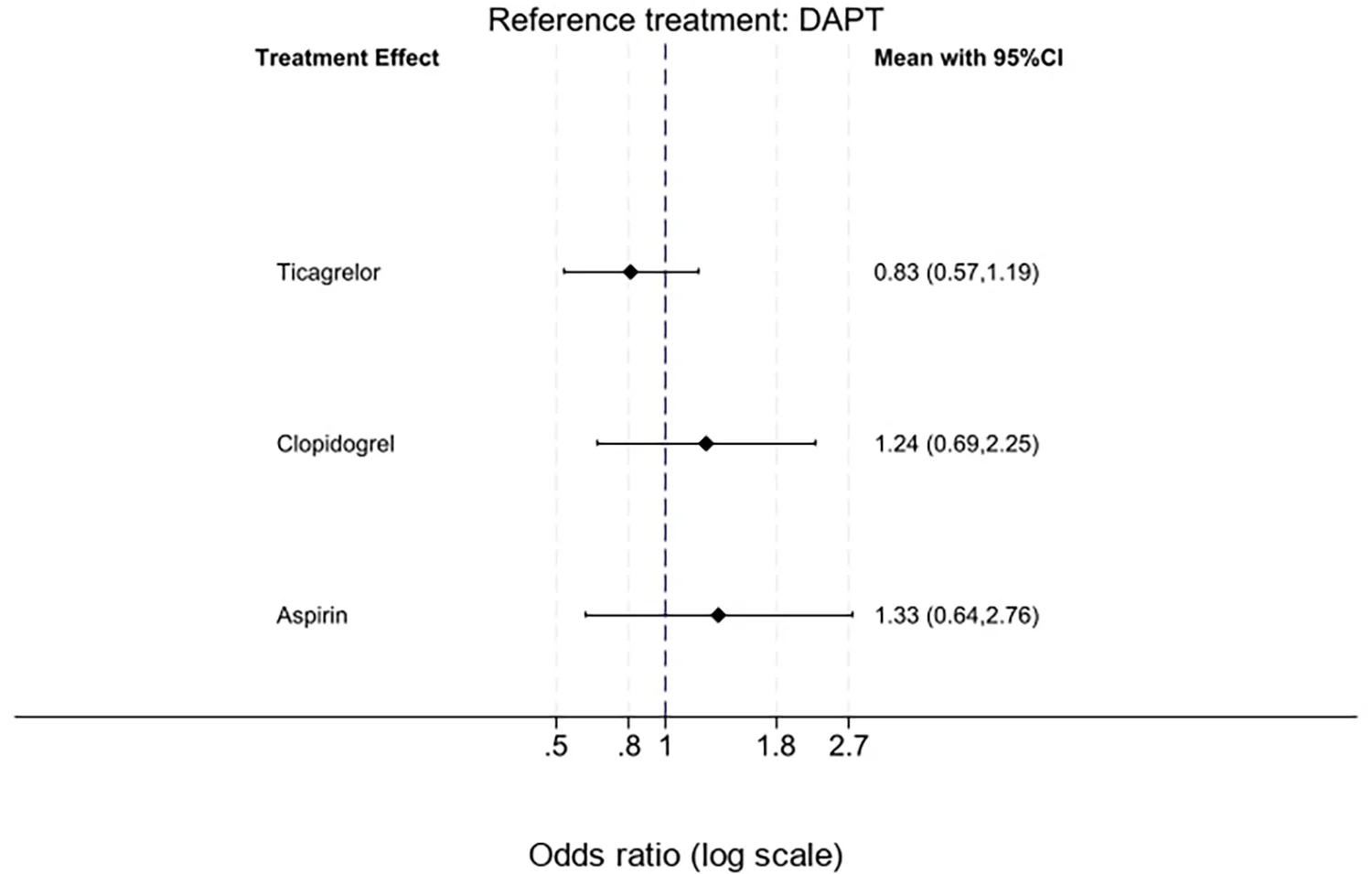
Forest plot for the primary efficacy outcome: major adverse cardiovascular events (MACE).

### 3.6 Myocardial infarction

For myocardial infarction, ticagrelor monotherapy did not differ significantly from DAPT (OR, 1.03; 95% CI, 0.69–1.56). In contrast, clopidogrel monotherapy and aspirin monotherapy were associated with higher risks of myocardial infarction compared with DAPT. The OR was 3.27 for clopidogrel monotherapy (95% CI, 1.29–8.28) and 3.80 for aspirin monotherapy (95% CI, 1.19–12.16) (Figure 7).

**Figure 7:**
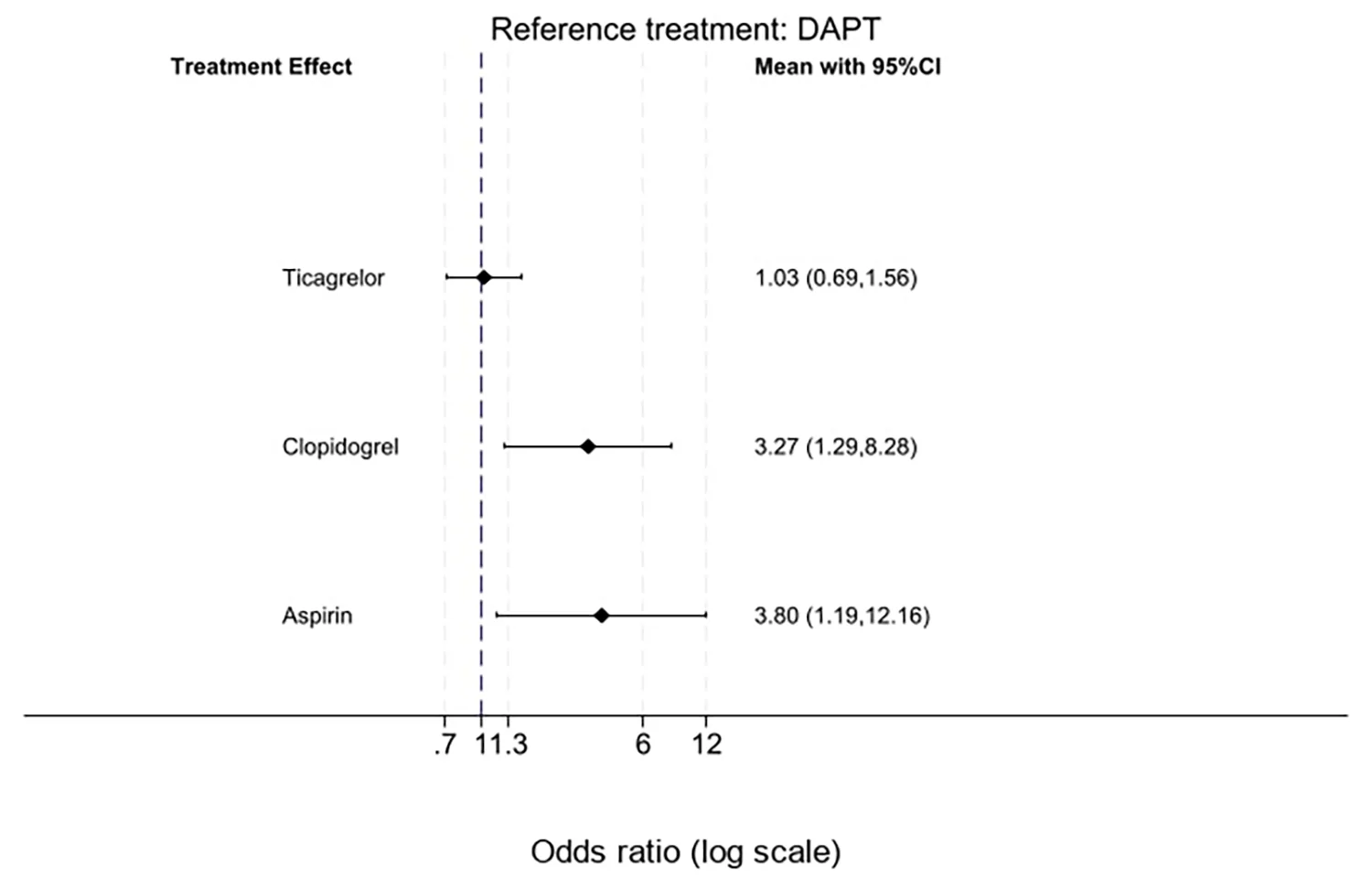
Forest plot for myocardial infarction.

### 3.7 All-cause death

No significant differences in all-cause death were observed between DAPT and any monotherapy strategy. Compared with DAPT, the OR was 0.76 for ticagrelor monotherapy (95% CI, 0.51–1.14), 1.29 for aspirin monotherapy (95% CI, 0.68–2.47), and 1.37 for clopidogrel monotherapy (95% CI, 0.79–2.38) (Figure 8).

**Figure 8:**
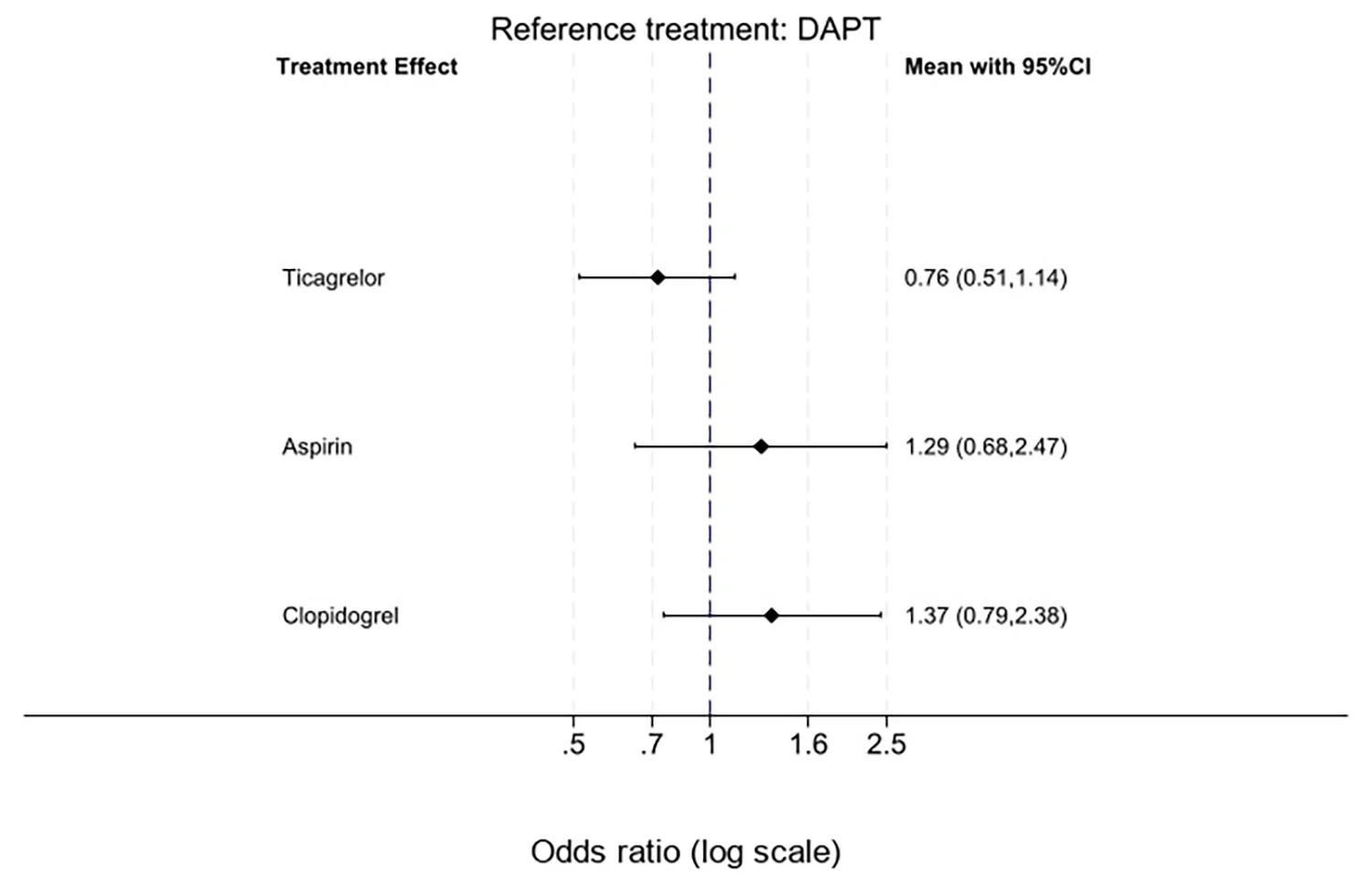
Forest plot for all-cause death.

### 3.8 Stroke

No significant differences in stroke were detected among the evaluated strategies. Compared with DAPT, the ORs were 0.93 for clopidogrel monotherapy (95% CI, 0.24–3.63), 0.99 for ticagrelor monotherapy (95% CI, 0.30–3.28), and 1.27 for aspirin monotherapy (95% CI, 0.20–7.97) (Figure 9). The confidence intervals were wide, indicating substantial imprecision.

**Figure 9:**
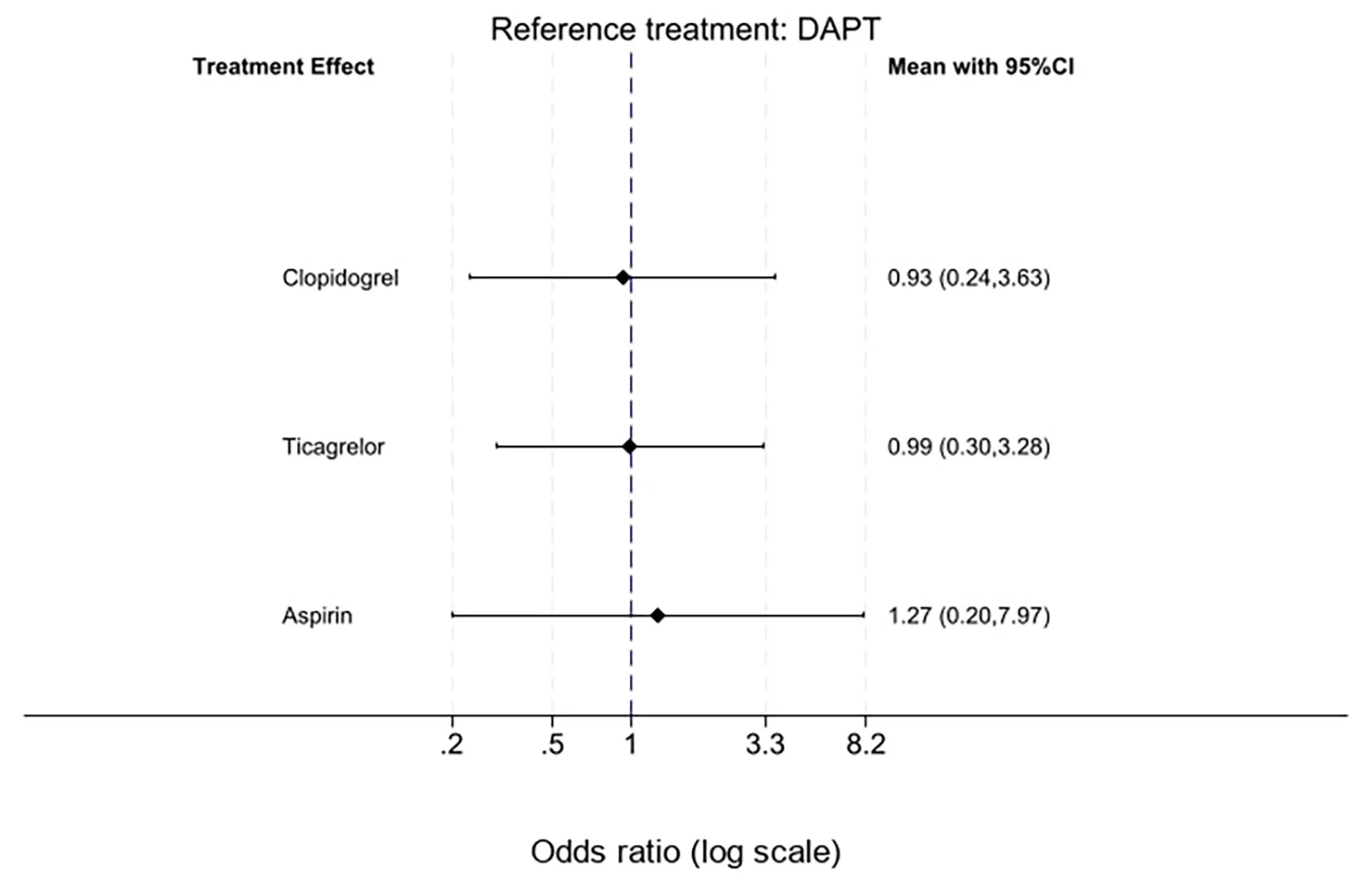
Forest plot for stroke.

### 3.9 Treatment ranking

Exploratory treatment rankings varied across outcomes. For major bleeding, aspirin monotherapy had the highest probability of ranking first and DAPT the highest probability of ranking last. Ticagrelor monotherapy ranked favorably for MACE and all-cause death. For myocardial infarction, DAPT and ticagrelor monotherapy ranked more favorably than aspirin or clopidogrel monotherapy. These MI rankings should not be taken as evidence of intrinsic harm from aspirin or clopidogrel. For stroke, ranking probabilities were dispersed, with few events and wide confidence intervals (Figures 10–14). The sparse network, absence of closed loops, and limited assessment of heterogeneity substantially restrict confidence in all of these rankings. They do not establish a reliable hierarchy of treatment efficacy or safety.

**Figure 10:**
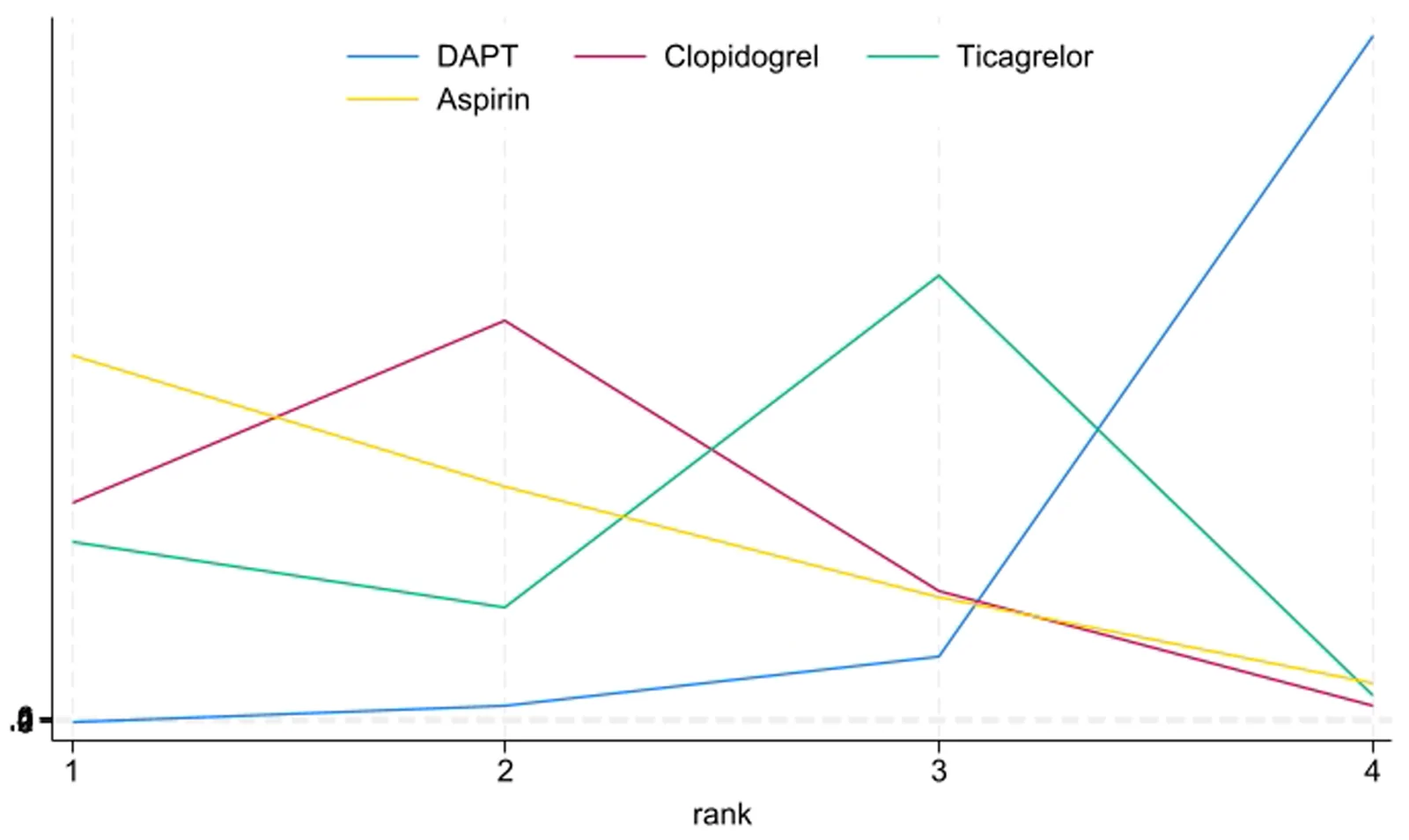
Cumulative ranking probability plot (SUCRA) for major bleeding.

**Figure 11:**
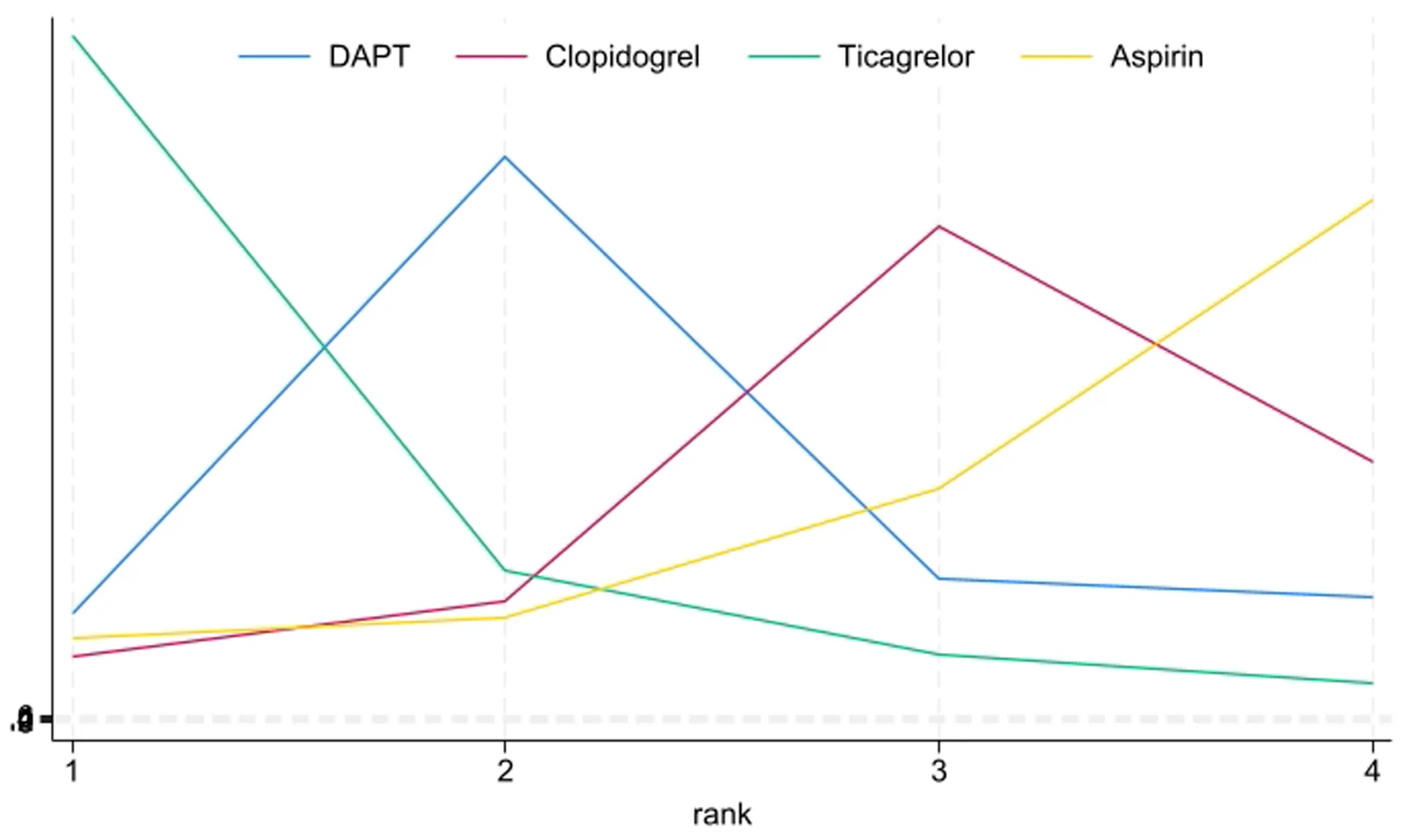
Cumulative ranking probability plot (SUCRA) for MACE.

**Figure 12:**
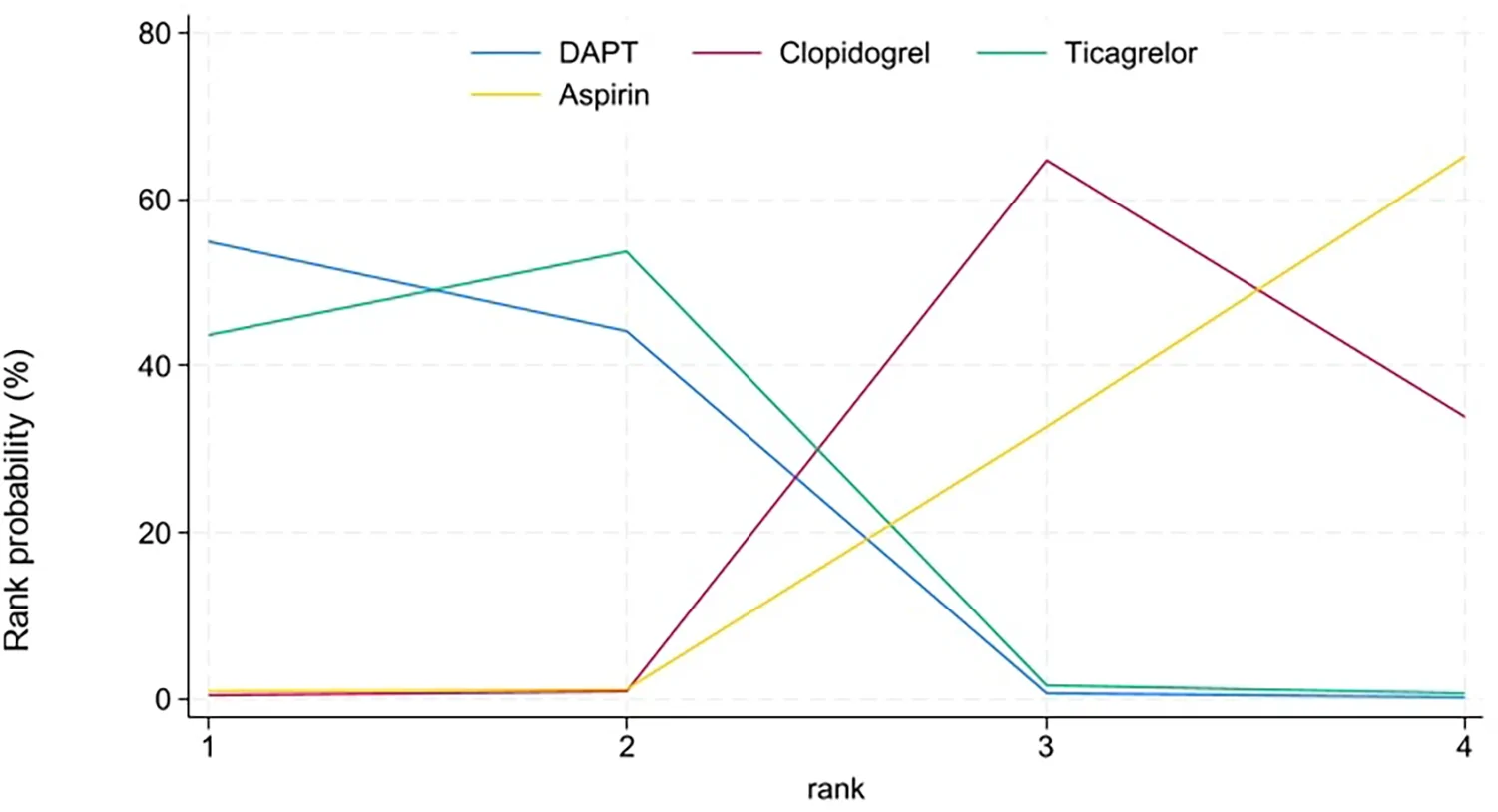
Cumulative ranking probability plot (SUCRA) for myocardial infarction.

**Figure 13:**
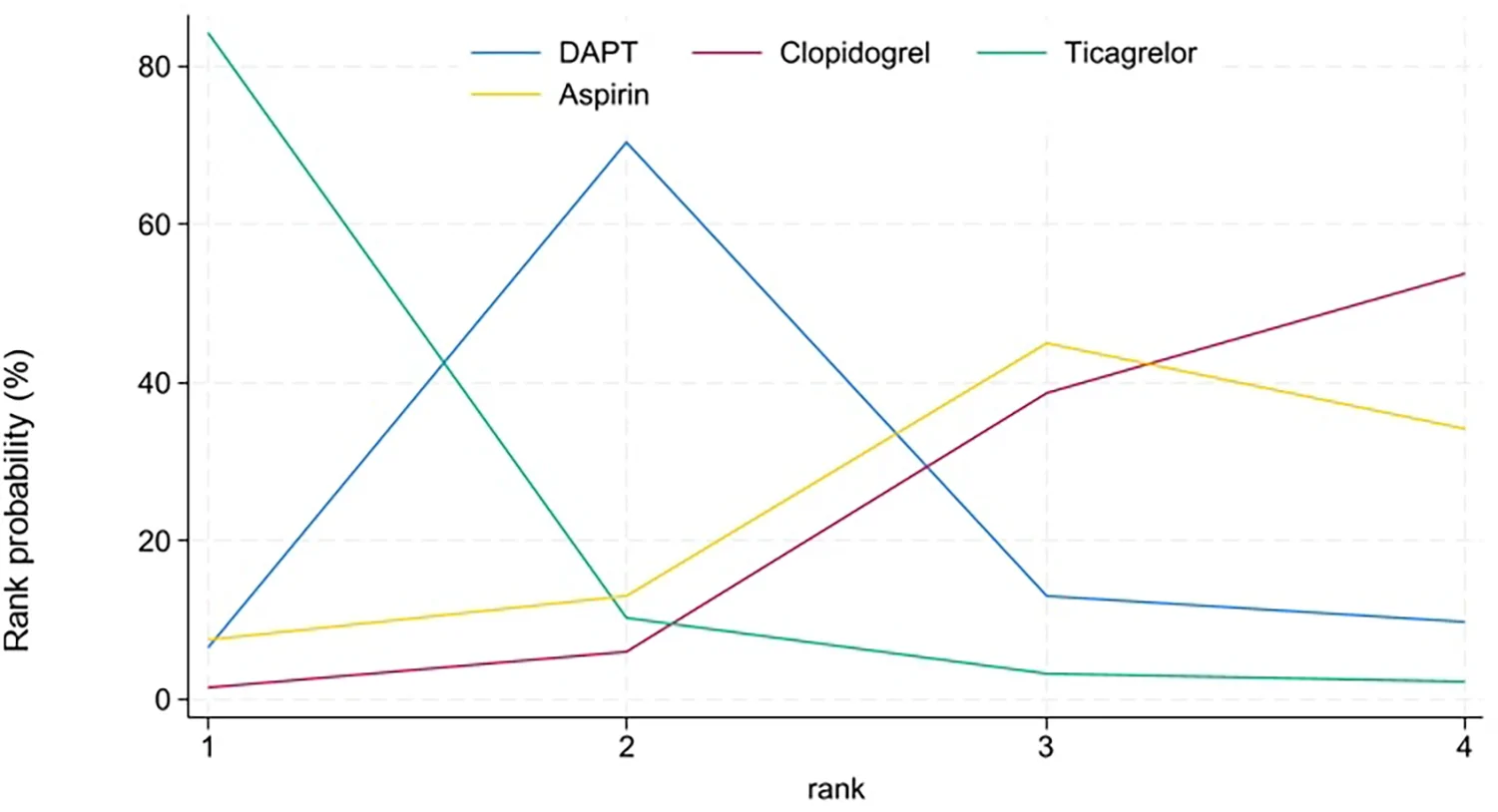
Cumulative ranking probability plot (SUCRA) for all-cause death.

**Figure 14:**
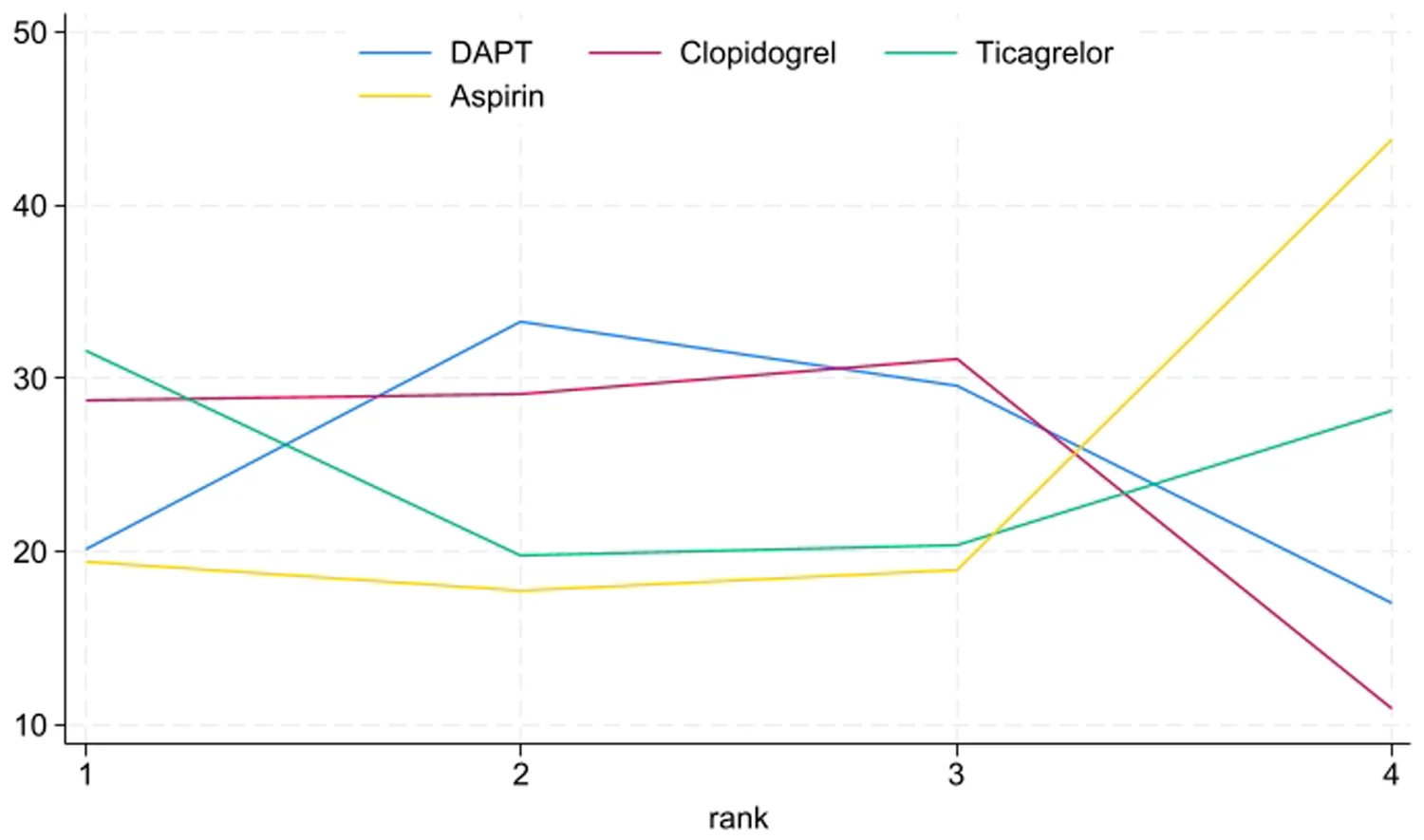
Cumulative ranking probability plot (SUCRA) for stroke.

### 3.10 Sensitivity analyses

Leave-one-out sensitivity analyses were performed by sequentially excluding individual studies. Estimates for all-cause death were highly stable after exclusion of individual studies. MACE estimates were generally stable, although some indirect comparisons changed in magnitude.

For myocardial infarction and stroke, the ticagrelor versus DAPT estimate was sensitive to the exclusion of Studies 2 and 3, with reversal of the direction of effect in some analyses. For major bleeding, the direction of most comparisons was preserved after exclusion of Studies 1 to 3. However, exclusion of Studies 4, 5, or 6 led to disconnected networks or non-estimable contrasts. These findings indicate that several estimates were influenced by the sparse network structure and should be interpreted cautiously (Figures 15–19).

**Figure 15:**
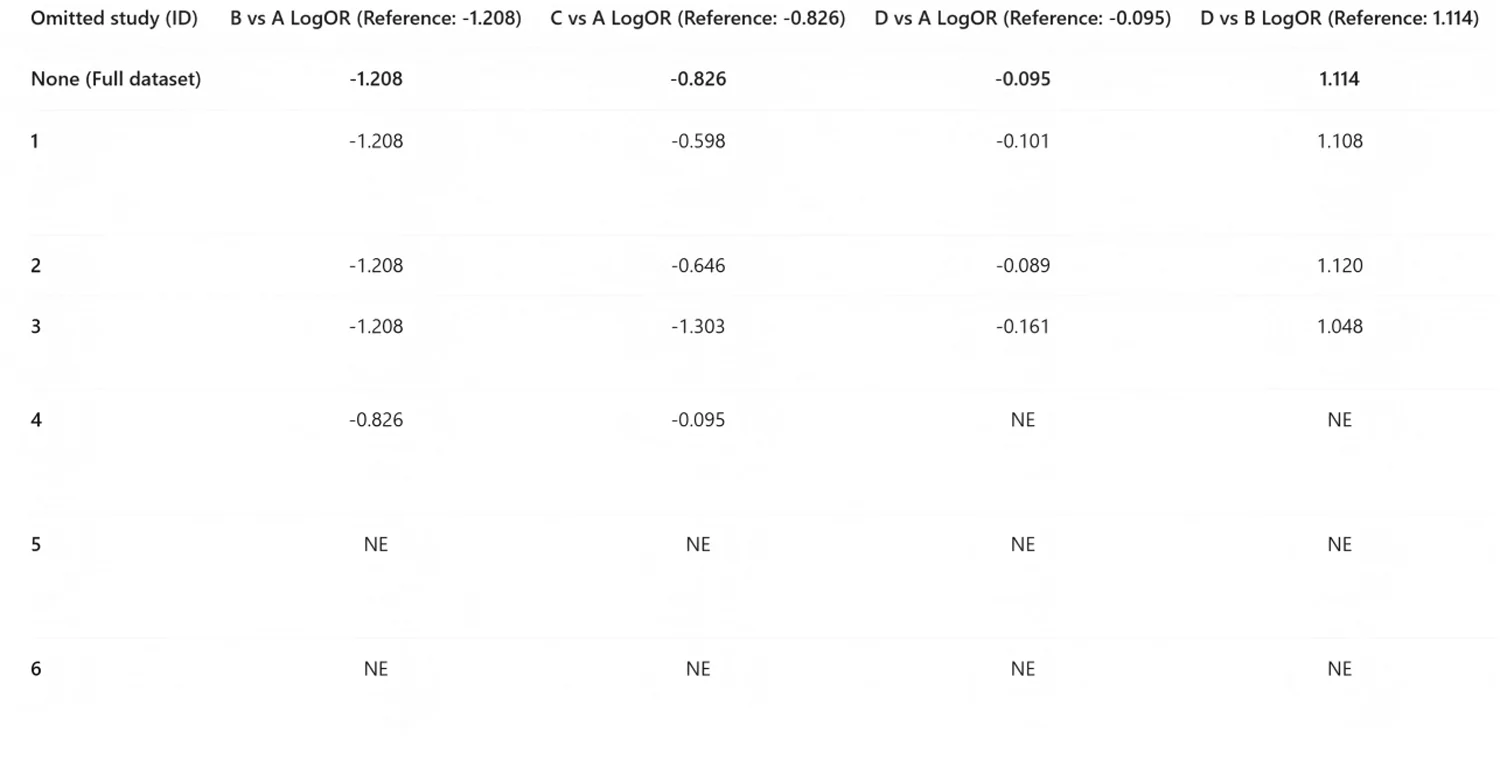
Leave-one-out sensitivity analysis for major bleeding.

**Figure 16:**
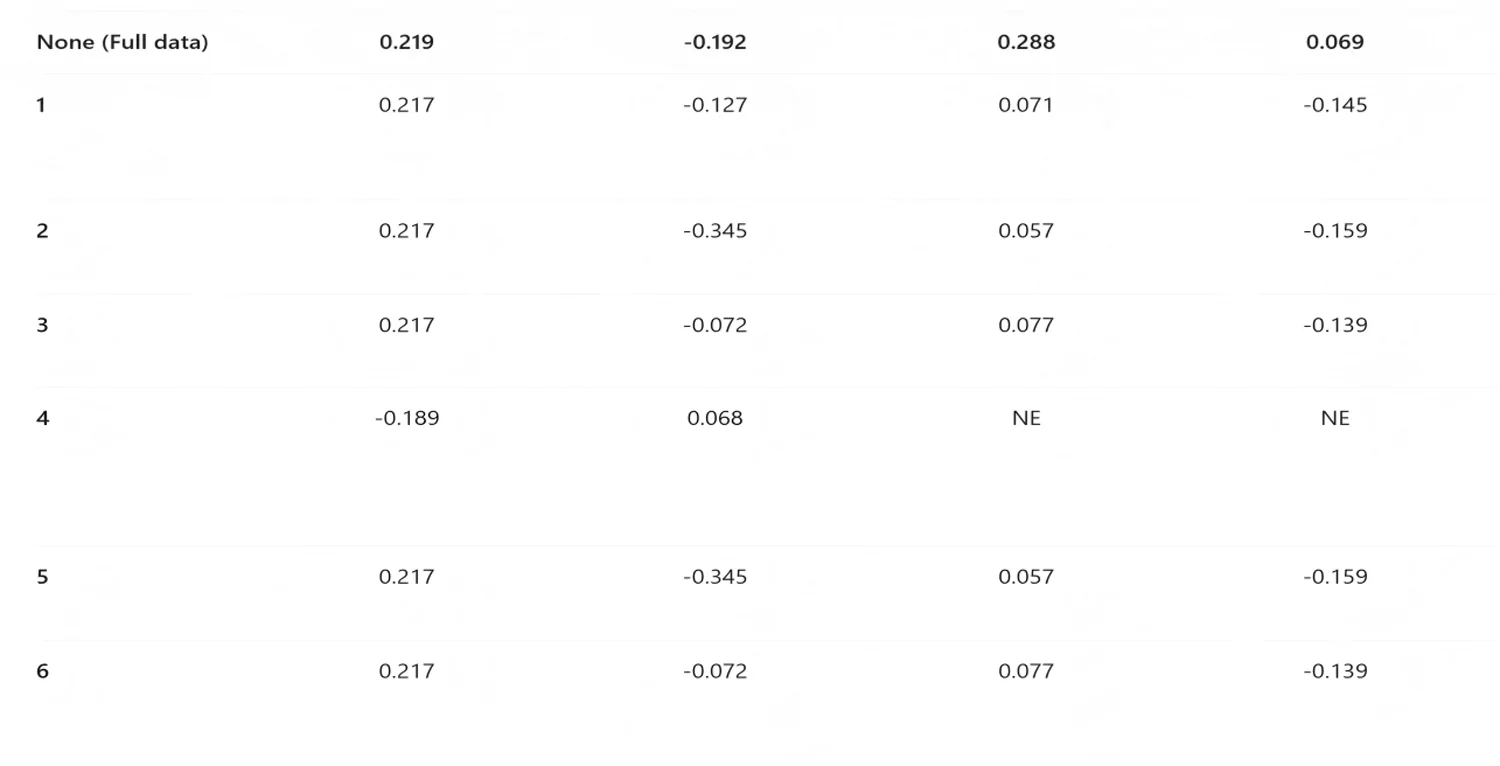
Leave-one-out sensitivity analysis for MACE.

**Figure 17:**
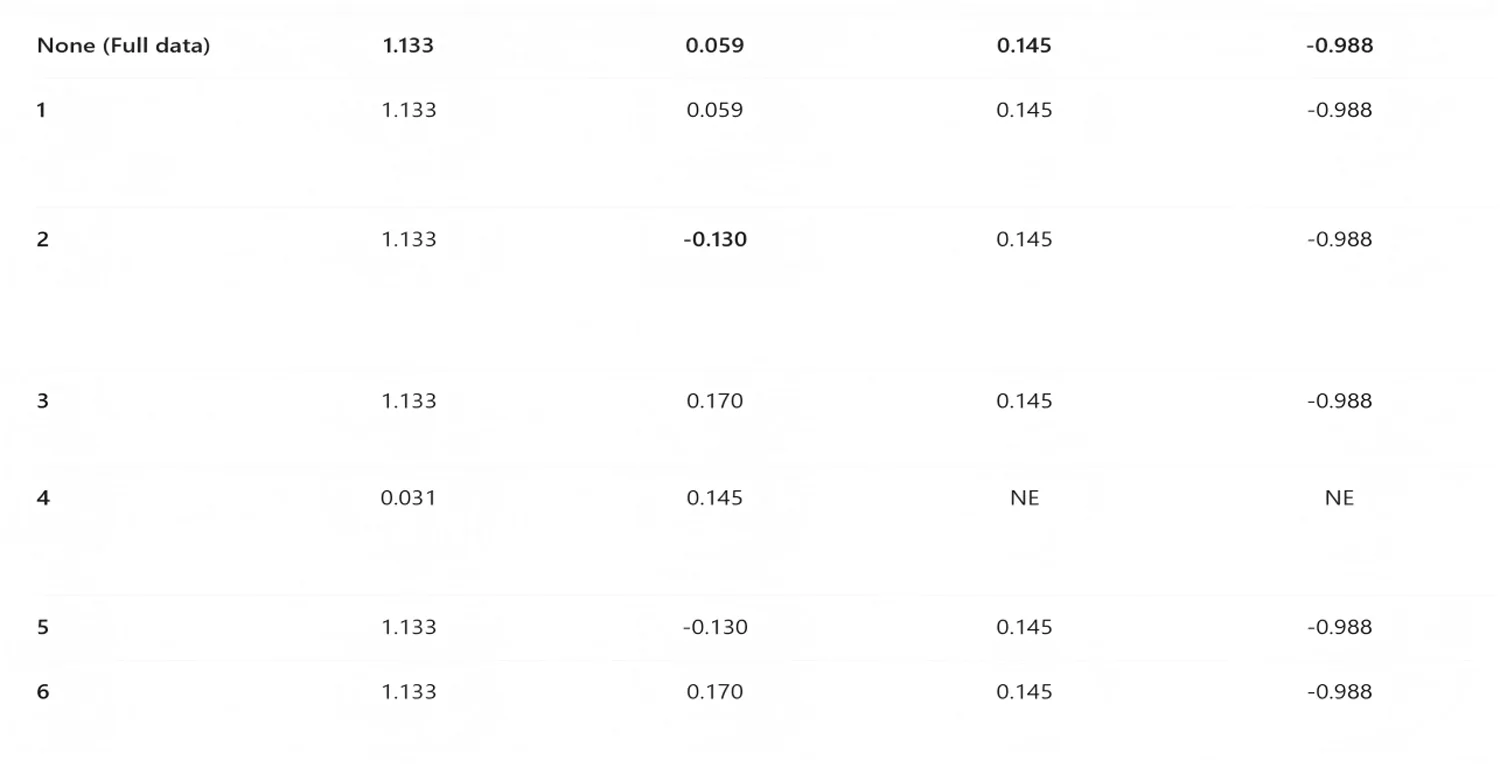
Leave-one-out sensitivity analysis for myocardial infarction.

**Figure 18:**
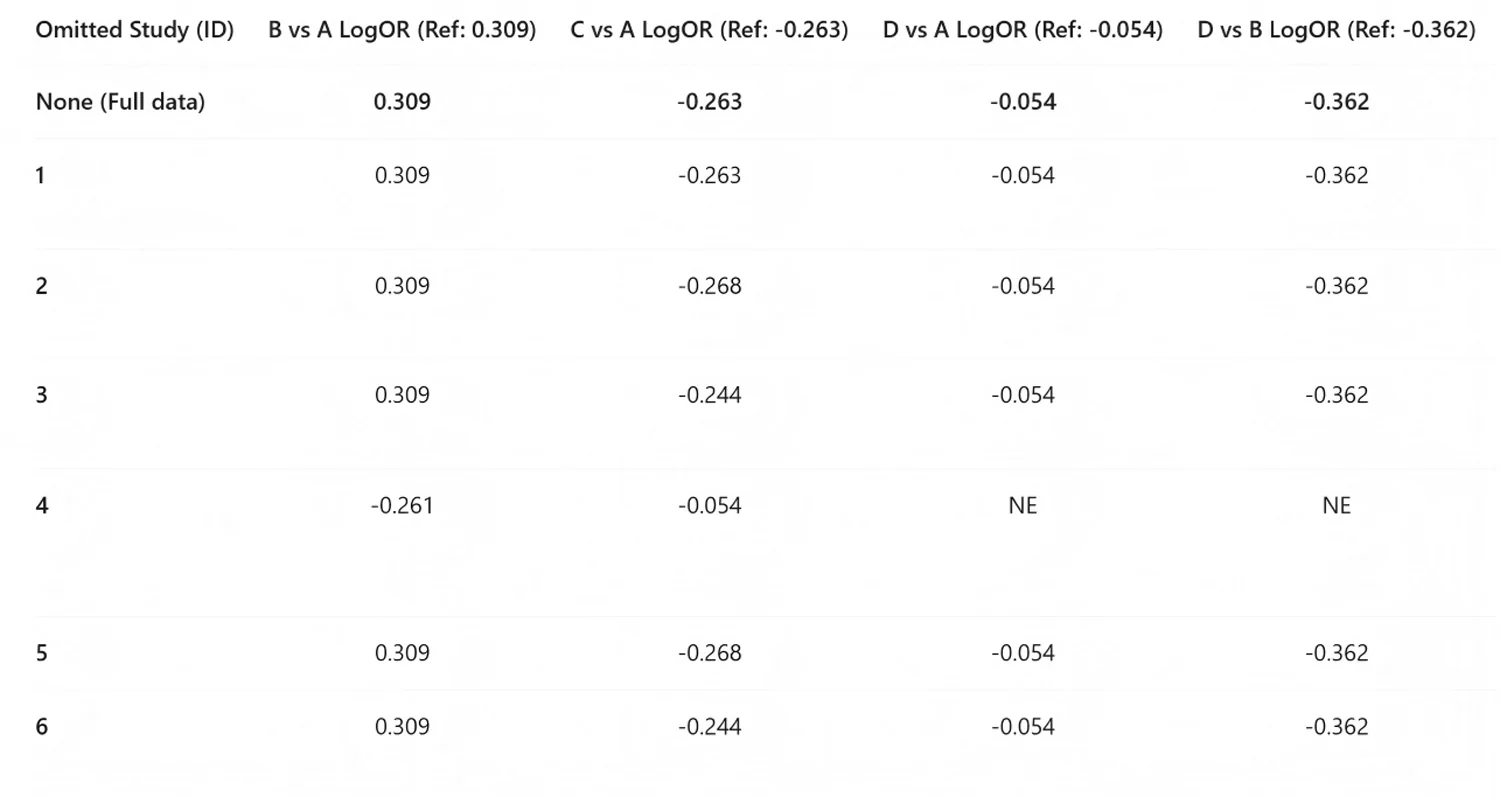
Leave-one-out sensitivity analysis for all-cause death.

**Figure 19:**
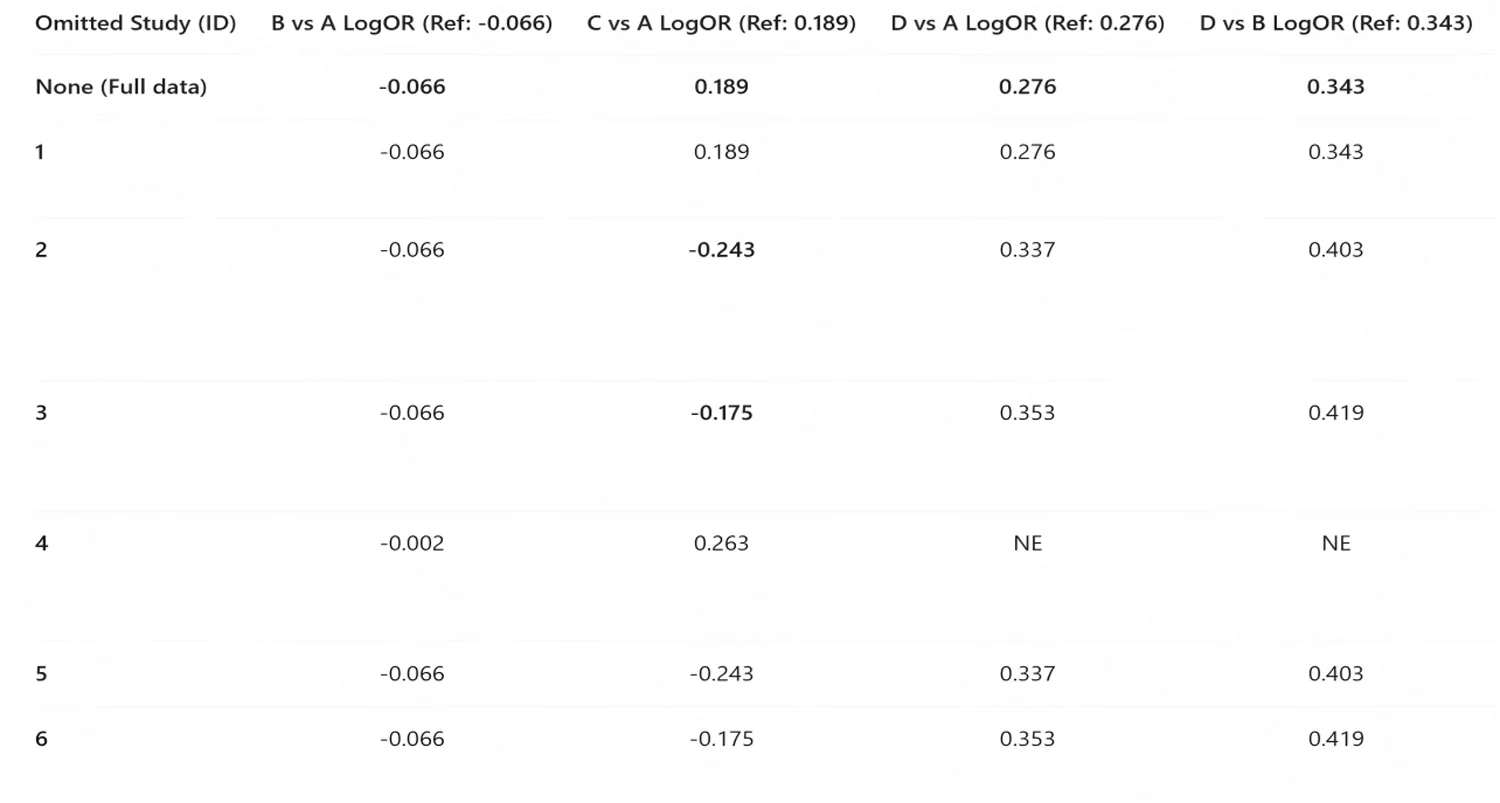
Leave-one-out sensitivity analysis for troke.

### 3.11 Certainty of evidence

CINeMA assessment indicated low certainty of evidence across outcomes and comparisons, mainly because of imprecision related to sparse evidence, wide confidence intervals, and wide prediction intervals. Additional concerns regarding heterogeneity or incoherence were present in selected comparisons. Detailed certainty assessments are provided in the Supplementary Materials.

## 4 Discussion

In this frequentist network meta-analysis of randomized evidence, we compared antiplatelet monotherapy maintenance strategies after an ultrashort course of DAPT in patients with coronary artery disease at high bleeding risk who were not receiving oral anticoagulant therapy. The main findings were as follows. First, aspirin, clopidogrel, and ticagrelor monotherapy showed numerically lower risks of major bleeding than DAPT, although none of these comparisons reached statistical significance. Second, no significant differences in MACE, all-cause death, or stroke were observed between DAPT and any monotherapy strategy. The higher MI estimates should be viewed as exploratory signals given the sparse network and the contribution of indirect evidence, rather than as evidence of intrinsic harm from either drug. Finally, the treatment rankings were exploratory and did not establish a reliable safety or efficacy hierarchy, given the sparse network and low certainty of evidence.

These numerical trends are consistent with several prior RCTs that examined specific monotherapy strategies in HBR or HBR-featured populations. In the TICO-HBR subanalysis, ticagrelor monotherapy after DAPT was associated with a lower rate of net adverse clinical events versus continued DAPT, with a consistent treatment effect across ARC-HBR and PRECISE-DAPT–defined HBR (16). Similarly, TWILIGHT-HBR showed that ticagrelor monotherapy after 3-month DAPT reduced BARC 2/3/5 bleeding by 47% and BARC 3/5 by 69% versus DAPT in HBR patients, without increasing ischemia (14). In the STOPDAPT-2 program, DAPT followed by clopidogrel monotherapy reduced TIMI major/minor bleeding versus 12-month DAPT in subgroups enriched for HBR features (19), and Obayashi et al. recently reported from STOPDAPT-3 that among patients with ACS and HBR, aspirin versus clopidogrel monotherapy after 1 month yielded no significant differences in cardiovascular or bleeding endpoints (12). Prior individual RCTs in HBR have also established that 1-month DAPT followed by P2Y12 monotherapy reduces bleeding versus 12-month DAPT (MASTER DAPT (20), STOPDAPT-2 main trial (21), with consistent signals across HBR-defined subgroups.

The reduction of bleeding risk is a central consideration in patients at high bleeding risk after PCI. In the present analysis, all monotherapy strategies had point estimates below unity for major bleeding compared with DAPT, consistent with the biological and clinical expectation that withdrawal of one antiplatelet agent may reduce bleeding exposure. Although all three point estimates were below 1, each confidence interval was wide and crossed 1. For aspirin monotherapy, the OR was 0.26, but the 95% CI extended from 0.05 to 1.42.

The ischemic findings require more careful interpretation. Although MACE did not differ significantly between DAPT and any monotherapy strategy, clopidogrel and aspirin monotherapy had higher estimated odds of MI than DAPT. Both 95% confidence intervals excluded unity, but they were wide, and the comparisons were informed by a sparse network that included indirect evidence. These findings should therefore be treated as signals requiring further investigation rather than evidence of intrinsic harm from either drug. The MI comparison for ticagrelor should not be interpreted as evidence of preserved ischemic protection or superiority over aspirin or clopidogrel. Longer-term maintenance evidence from HOST-EXAM has favored clopidogrel over aspirin for composite clinical outcomes (11). The recent meta-analysis by Ibrahim et al. also reported fewer major adverse cardiac and cerebrovascular events with clopidogrel than with aspirin (22). However, these maintenance-treatment comparisons address a different question from monotherapy versus continued DAPT after abbreviated DAPT in HBR patients. They neither directly resolve nor negate the MI signals observed here. Given the low certainty of evidence, these signals should be considered hypothesis-generating and investigated in adequately powered direct comparisons.

In the present analysis, ticagrelor monotherapy showed numerically favorable estimates for MACE and major bleeding compared with DAPT, but neither comparison reached statistical significance. These ischemic findings are consistent with the parent TICO (16)and TWILIGHT (14) trials, both of which showed non-inferiority or superiority of ticagrelor monotherapy versus DAPT for net clinical outcomes. An earlier individual patient data meta-analysis compared P2Y12 inhibitor with aspirin monotherapy (23). The estimates for major bleeding (OR, 0.42; 95% CI, 0.17–1.08) and MACE (OR, 0.83; 95% CI, 0.57–1.19) remained imprecise. The absence of a statistically significant difference in MI does not establish preserved ischemic protection. These results do not demonstrate a superior ischemic and bleeding balance for ticagrelor monotherapy.

Recent meta-analyses provide complementary evidence on antiplatelet monotherapy after PCI. Giacoppo et al. reported fewer major adverse cardiac and cerebrovascular events with P2Y12 inhibitor monotherapy than with aspirin, with no significant difference in major bleeding (24). Ibrahim et al. reported similar findings for clopidogrel versus aspirin, although their analysis included both randomized and observational studies (22). Both reviews included bleeding-risk subgroup analyses, but their overall estimates were derived from broader PCI populations and long-term maintenance-treatment settings. Differences in patient selection and treatment timing should be considered when comparing their findings with our analysis of monotherapy following abbreviated DAPT in HBR patients.

All-cause death and stroke did not differ significantly across treatment strategies. These outcomes were relatively infrequent, particularly stroke, leading to wide confidence intervals and limited statistical precision. Therefore, the absence of significant differences should not be interpreted as evidence of equivalence. Rather, the available randomized evidence remains insufficient to determine whether any monotherapy strategy materially changes the risk of death or stroke in this population.

These observations sit alongside current guideline recommendations that HBR patients may transition to SAPT after 1–3 months of DAPT (25–27), although the optimal lead-in duration and choice of SAPT agent in truly ultrashort DAPT contexts remain to be defined.

This study has several strengths. We focused on randomized evidence and restricted the population to high bleeding risk patients not receiving oral anticoagulant therapy, thereby reducing clinical heterogeneity related to anticoagulant-based or triple antithrombotic regimens. We also evaluated both bleeding and ischemic outcomes at 1 year, which is clinically relevant for assessing maintenance antiplatelet strategies after PCI. In addition, we used CINeMA to assess the certainty of network estimates and performed leave-one-out sensitivity analyses to examine the influence of individual studies.

Several limitations should be acknowledged. First, only six studies contributed to the network. The absence of closed loops precluded formal inconsistency testing, while the small number of studies and limited evidence within treatment comparisons prevented reliable assessment of between-study heterogeneity. These limitations substantially reduce confidence in the comparative rankings. Second, although a random-effects model was used, the heterogeneity variance was estimated from only six studies. The confidence intervals were based on normal approximations and may not fully reflect uncertainty in the estimated heterogeneity variance. Random-effects modeling also does not remove clinical or methodological differences between studies. The treatment estimates and rankings should therefore be interpreted cautiously. Third, the analysis included both original randomized trials and subgroup analyses of randomized trials, which may differ in statistical power and patient selection. Fourth, although all included patients were at high bleeding risk and were not receiving oral anticoagulant therapy, definitions of high bleeding risk were based on the original studies and may not have been fully uniform. Fifth, the certainty of evidence was low across outcomes and comparisons, mainly due to imprecision and sparse evidence. Finally, treatment rankings were exploratory and should not be used alone to guide clinical decision-making.

In summary, among high bleeding risk patients undergoing PCI who were not receiving oral anticoagulant therapy, antiplatelet monotherapy after ultrashort DAPT showed a possible reduction in major bleeding compared with DAPT, but the estimates were not statistically significant. Ticagrelor monotherapy showed numerically favorable estimates for MACE and major bleeding compared with DAPT, without statistically significant differences. Aspirin monotherapy ranked favorably for major bleeding. Given the sparse network and low certainty of evidence, these findings should be interpreted cautiously and should be confirmed in adequately powered randomized trials focused specifically on high bleeding risk patients.

## 5 Conclusions

In patients with coronary artery disease at high bleeding risk who were not receiving oral anticoagulant therapy, the available evidence was insufficient to determine whether antiplatelet monotherapy after an ultrashort course of DAPT reduces major bleeding compared with DAPT.No significant differences were observed for MACE, all-cause death, or stroke across the evaluated strategies. For MI, the ORs for aspirin and clopidogrel monotherapy versus DAPT were above 1, with confidence intervals excluding 1. However, the wide intervals and sparse network limit confidence in these estimates. The ticagrelor estimate was inconclusive and does not establish equivalence to DAPT.

Ticagrelor monotherapy showed numerically favorable estimates for MACE and major bleeding compared with DAPT, but neither comparison reached statistical significance. These findings do not establish a superior ischemic and bleeding balance. The sparse network, absence of closed loops, and low certainty of evidence preclude firm conclusions about the relative ranking of the monotherapy strategies. Further adequately powered randomized trials specifically enrolling high bleeding risk patients after PCI are needed to clarify the optimal antiplatelet monotherapy strategy after ultrashort DAPT.

## Data Availability

The original contributions presented in the study are included in the article/Supplementary Material, further inquiries can be directed to the corresponding author.
