# Supplementary material for: Antiplatelet monotherapy maintenance strategies after percutaneous coronary intervention in patients with coronary artery disease at high bleeding risk: a network meta-analysis
Source: Front Cardiovasc Med. 2026 Oct 28;13:1941814. doi: 10.3389/fcvm.2026.1941814 (PMC13751440; doi:10.3389/fcvm.2026.1941814)
Supplement: Supplementary file 1 [file Datasheet1.pdf]

Confidence In Network Meta Analysis - CINeMA 2.0.0 - Project: **All-cause death**

All-cause death

Comparison	Number of Studies	Within-study bias	Reporting bias	Indirectness	Imprecision	Heterogeneity	Incoherence	Confidence rating	Reason(s) for downgrading
Mixed evidence									
**Aspirin vs Clopidogrel**	2	No concerns<br>**Majority RoB:** No concerns<br>**Average RoB:** No concerns (applied)<br>**Highest RoB:** No concerns	Low risk<br>**Evidence:** mixed<br>**Source:** manual	No concerns<br>**Majority:** low<br>**Average:** low<br>**Highest:** low	Major concerns<br>**NMA effect:** 0.947<br>**95% CI:** (0.679, 1.320)<br>crosses 2 clinically important line(s)<br>**Clinically important:** (0.8, 1.25)	No concerns<br>**NMA effect:** 0.947<br>**95% CI:** (0.679, 1.320)<br>**95% PrI:** (0.552, 1.624)<br>**τ^2:** —<br>**I^2:** null%	No concerns<br>**Evidence:** direct only<br>IF not applicable; judged from the design-by-treatment test.	Low	|

Comparison	Number of Studies	Within-study bias	Reporting bias	Indirectness	Imprecision	Heterogeneity	Incoherence	Confidence rating	Reason(s) for downgrading
**Clopidogrel vs Standard treatment**	1	No concerns<br>**Majority RoB:** No concerns<br>**Average RoB:** No concerns (applied)<br>**Highest RoB:** No concerns	Low risk<br>**Evidence:** mixed<br>**Source:** manual	No concerns<br>**Majority:** low<br>**Average:** low<br>**Highest:** low	Major concerns<br>**NMA effect:** 1.368<br>**95% CI:** (0.785, 2.383)<br>crosses 2 clinically important line(s)<br>**Clinically important:** (0.8, 1.25)	No concerns<br>**NMA effect:** 1.368<br>**95% CI:** (0.785, 2.383)<br>**95% PrI:** (0.556, 3.368)<br>**τ^2:** —<br>**I^2:** null%	No concerns<br>**Evidence:** direct only<br>IF not applicable; judged from the design-by-treatment test.	Low	|
**Standard treatment vs Ticagrelor**	3	No concerns<br>**Majority RoB:** No concerns<br>**Average RoB:** No concerns (applied)<br>**Highest RoB:** No concerns	Low risk<br>**Evidence:** mixed<br>**Source:** manual	No concerns<br>**Majority:** low<br>**Average:** low<br>**Highest:** low	Some concerns<br>**NMA effect:** 1.309<br>**95% CI:** (0.880, 1.949)<br>crosses 1 clinically important line(s)<br>**Clinically important:** (0.8, 1.25)	Some concerns<br>**NMA effect:** 1.309<br>**95% CI:** (0.880, 1.949)<br>**95% PrI:** (0.686, 2.498)<br>**τ^2:** 0.000<br>**I^2:** 0.0%%	No concerns<br>**Evidence:** direct only<br>IF not applicable; judged from the design-by-treatment test.	Low	|

Indirect evidence

Comparison	Number of Studies	Within-study bias	Reporting bias	Indirectness	Imprecision	Heterogeneity	Incoherence	Confidence rating	Reason(s) for downgrading
**Aspirin vs Standard treatment**	--	No concerns<br>**Majority RoB:** No concerns<br>**Average RoB:** No concerns (applied)<br>**Highest RoB:** No concerns	Low risk<br>**Evidence:** mixed<br>**Source:** manual	No concerns	Major concerns<br>**NMA effect:** 1.295<br>**95% CI:** (0.678, 2.473)<br>crosses 2 clinically important line(s)<br>**Clinically important:** (0.8, 1.25)	No concerns<br>**NMA effect:** 1.295<br>**95% CI:** (0.678, 2.473)<br>**95% PrI:** (0.453, 3.701)	No concerns<br>**Evidence:** indirect only<br>IF not applicable; judged from the design-by-treatment test.	Low	
**Aspirin vs Ticagrelor**	--	No concerns<br>**Majority RoB:** No concerns<br>**Average RoB:** No concerns (applied)<br>**Highest RoB:** No concerns	Low risk<br>**Evidence:** mixed<br>**Source:** manual	No concerns	Major concerns<br>**NMA effect:** 1.696<br>**95% CI:** (0.794, 3.623)<br>crosses 2 clinically important line(s)<br>**Clinically important:** (0.8, 1.25)	No concerns<br>**NMA effect:** 1.696<br>**95% CI:** (0.794, 3.623)<br>**95% PrI:** (0.494, 5.817)	No concerns<br>**Evidence:** indirect only<br>IF not applicable; judged from the design-by-treatment test.	Low	

Comparison	Number of Studies	Within-study bias	Reporting bias	Indirectness	Imprecision	Heterogeneity	Incoherence	Confidence rating	Reason(s) for downgrading
Clopidogrel vs Ticagrelor	--	No concerns<br>**Majority RoB:** No concerns<br>**Average RoB:** No concerns (applied)<br>**Highest RoB:** No concerns	Low risk<br>**Evidence:** mixed<br>**Source:** manual	No concerns	Some concerns<br>**NMA effect:** 1.791<br>**95% CI:** (0.905, 3.545)<br>crosses 1 clinically important line(s)<br>**Clinically important:** (0.8, 1.25)	Some concerns<br>**NMA effect:** 1.791<br>**95% CI:** (0.905, 3.545)<br>**95% PrI:** (0.591, 5.427)	No concerns<br>**Evidence:** indirect only<br>IF not applicable; judged from the design-by-treatment test.	Low	|
