# Supplementary material for: Antiplatelet monotherapy maintenance strategies after percutaneous coronary intervention in patients with coronary artery disease at high bleeding risk: a network meta-analysis
Source: Front Cardiovasc Med. 2026 Oct 28;13:1941814. doi: 10.3389/fcvm.2026.1941814 (PMC13751440; doi:10.3389/fcvm.2026.1941814)
Supplement: Supplementary file 2 [file Datasheet2.pdf]

Confidence In Network Meta Analysis - CINeMA 2.0.0 - Project: **MACE**

MACE

Comparison	Number of Studies	Within-study bias	Reporting bias	Indirectness	Imprecision	Heterogeneity	Incoherence	Confidence rating	Reason(s) for downgrading
Mixed evidence									
**Aspirin vs Clopidogrel**	2	No concerns<br>**Majority RoB:** No concerns<br>**Average RoB:** No concerns (applied)<br>**Highest RoB:** No concerns	Low risk<br>**Evidence:** mixed<br>**Source:** manual	No concerns<br>**Majority:** low<br>**Average:** low<br>**Highest:** low	Major concerns<br>**NMA effect:** 1.074<br>**95% CI:** (0.701, 1.644)<br>crosses 2 clinically important line(s)<br>**Clinically important:** (0.8, 1.25)	No concerns<br>**NMA effect:** 1.074<br>**95% CI:** (0.701, 1.644)<br>**95% PrI:** (0.421, 2.737)<br>**τ^2:** —<br>**I^2:** null%	No concerns<br>**Evidence:** direct only<br>IF not applicable; judged from the design-by-treatment test.	Low	

Comparison	Number of Studies	Within-study bias	Reporting bias	Indirectness	Imprecision	Heterogeneity	Incoherence	Confidence rating	Reason(s) for downgrading
**Clopidogrel vs Standard treatment**	1	No concerns<br>**Majority RoB:** No concerns<br>**Average RoB:** No concerns (applied)<br>**Highest RoB:** No concerns	Low risk<br>**Evidence:** mixed<br>**Source:** manual	No concerns<br>**Majority:** low<br>**Average:** low<br>**Highest:** low	Major concerns<br>**NMA effect:** 1.244<br>**95% CI:** (0.677, 2.287)<br>crosses 2 clinically important line(s)<br>**Clinically important:** (0.8, 1.25)	No concerns<br>**NMA effect:** 1.244<br>**95% CI:** (0.677, 2.287)<br>**95% PrI:** (0.385, 4.017)<br>**τ^2:** —<br>**I^2:** null%	No concerns<br>**Evidence:** direct only<br>IF not applicable; judged from the design-by-treatment test.	Low	|
**Standard treatment vs Ticagrelor**	3	No concerns<br>**Majority RoB:** No concerns<br>**Average RoB:** No concerns (applied)<br>**Highest RoB:** No concerns	Low risk<br>**Evidence:** mixed<br>**Source:** manual	No concerns<br>**Majority:** low<br>**Average:** low<br>**Highest:** low	Some concerns<br>**NMA effect:** 1.212<br>**95% CI:** (0.831, 1.767)<br>crosses 1 clinically important line(s)<br>**Clinically important:** (0.8, 1.25)	Some concerns<br>**NMA effect:** 1.212<br>**95% CI:** (0.831, 1.767)<br>**95% PrI:** (0.503, 2.918)<br>**τ^2:** 0.038<br>**I^2:** 36.7%%	No concerns<br>**Evidence:** direct only<br>IF not applicable; judged from the design-by-treatment test.	Low	|

Indirect evidence

Comparison	Number of Studies	Within-study bias	Reporting bias	Indirectness	Imprecision	Heterogeneity	Incoherence	Confidence rating	Reason(s) for downgrading
Aspirin vs Standard treatment	--	No concerns<br>**Majority RoB:** No concerns<br>**Average RoB:** No concerns (applied)<br>**Highest RoB:** No concerns	Low risk<br>**Evidence:** mixed<br>**Source:** manual	No concerns	Major concerns<br>**NMA effect:** 1.336<br>**95% CI:** (0.636, 2.809)<br>crosses 2 clinically important line(s)<br>**Clinically important:** (0.8, 1.25)	No concerns<br>**NMA effect:** 1.336<br>**95% CI:** (0.636, 2.809)<br>**95% PrI:** (0.343, 5.210)	No concerns<br>**Evidence:** indirect only<br>IF not applicable; judged from the design-by-treatment test.	Low	
Aspirin vs Ticagrelor	--	No concerns<br>**Majority RoB:** No concerns<br>**Average RoB:** No concerns (applied)<br>**Highest RoB:** No concerns	Low risk<br>**Evidence:** mixed<br>**Source:** manual	No concerns	Major concerns<br>**NMA effect:** 1.619<br>**95% CI:** (0.704, 3.725)<br>crosses 2 clinically important line(s)<br>**Clinically important:** (0.8, 1.25)	No concerns<br>**NMA effect:** 1.619<br>**95% CI:** (0.704, 3.725)<br>**95% PrI:** (0.364, 7.201)	No concerns<br>**Evidence:** indirect only<br>IF not applicable; judged from the design-by-treatment test.	Low	

Comparison	Number of Studies	Within-study bias	Reporting bias	Indirectness	Imprecision	Heterogeneity	Incoherence	Confidence rating	Reason(s) for downgrading
**Clopidogrel vs Ticagrelor**	--	No concerns<br>**Majority RoB:** No concerns<br>**Average RoB:** No concerns (applied)<br>**Highest RoB:** No concerns	Low risk<br>**Evidence:** mixed<br>**Source:** manual	No concerns	Major concerns<br>**NMA effect:** 1.508<br>**95% CI:** (0.737, 3.085)<br>crosses 2 clinically important line(s)<br>**Clinically important:** (0.8, 1.25)	No concerns<br>**NMA effect:** 1.508<br>**95% CI:** (0.737, 3.085)<br>**95% PrI:** (0.402, 5.658)	No concerns<br>**Evidence:** indirect only<br>IF not applicable; judged from the design-by-treatment test.	Low	
