# Supplementary material for: Antiplatelet monotherapy maintenance strategies after percutaneous coronary intervention in patients with coronary artery disease at high bleeding risk: a network meta-analysis
Source: Front Cardiovasc Med. 2026 Oct 28;13:1941814. doi: 10.3389/fcvm.2026.1941814 (PMC13751440; doi:10.3389/fcvm.2026.1941814)
Supplement: Supplementary file 3 [file Datasheet3.pdf]

Confidence In Network Meta Analysis - CINeMA 2.0.0 - Project: **Major bleeding**

Major bleeding

Comparison	Number of Studies	Within-study bias	Reporting bias	Indirectness	Imprecision	Heterogeneity	Incoherence	Confidence rating	Reason(s) for downgrading
				Mixed evidence					
**Aspirin vs Clopidogrel**	2	No concerns<br>**Majority RoB:** No concerns<br>**Average RoB:** No concerns (applied)<br>**Highest RoB:** No concerns	Low risk<br>**Evidence:** mixed<br>**Source:** manual	No concerns<br>**Majority:** low<br>**Average:** low<br>**Highest:** low	Major concerns<br>**NMA effect:** 0.910<br>**95% CI:** (0.342, 2.425)<br>crosses 2 clinically important line(s)<br>**Clinically important:** (0.8, 1.25)	No concerns<br>**NMA effect:** 0.910<br>**95% CI:** (0.342, 2.425)<br>**95% PrI:** (0.079, 10.468)<br>**τ^2:** —<br>**I^2:** null%	Major concerns<br>**Evidence:** direct only<br>IF not applicable; judged from the design-by-treatment test.	Low	|

Comparison	Number of Studies	Within-study bias	Reporting bias	Indirectness	Imprecision	Heterogeneity	Incoherence	Confidence rating	Reason(s) for downgrading
**Clopidogrel vs Standard treatment**	1	No concerns<br>**Majority RoB:** No concerns<br>**Average RoB:** No concerns (applied)<br>**Highest RoB:** No concerns	Low risk<br>**Evidence:** mixed<br>**Source:** manual	No concerns<br>**Majority:** low<br>**Average:** low<br>**Highest:** low	Some concerns<br>**NMA effect:** 0.284<br>**95% CI:** (0.069, 1.172)<br>crosses 1 clinically important line(s)<br>**Clinically important:** (0.8, 1.25)	Some concerns<br>**NMA effect:** 0.284<br>**95% CI:** (0.069, 1.172)<br>**95% PrI:** (0.015, 5.449)<br>**τ²:** —<br>**I²:** null%	Major concerns<br>**Evidence:** direct only<br>IF not applicable; judged from the design-by-treatment test.	Low	
**Standard treatment vs Ticagrelor**	3	No concerns<br>**Majority RoB:** No concerns<br>**Average RoB:** No concerns (applied)<br>**Highest RoB:** No concerns	Low risk<br>**Evidence:** mixed<br>**Source:** manual	No concerns<br>**Majority:** low<br>**Average:** low<br>**Highest:** low	Some concerns<br>**NMA effect:** 2.376<br>**95% CI:** (0.979, 5.766)<br>crosses 1 clinically important line(s)<br>**Clinically important:** (0.8, 1.25)	Some concerns<br>**NMA effect:** 2.376<br>**95% CI:** (0.979, 5.766)<br>**95% PrI:** (0.227, 24.824)<br>**τ²:** 0.635<br>**I²:** 71.7%%	Major concerns<br>**Evidence:** direct only<br>IF not applicable; judged from the design-by-treatment test.	Low	

Indirect evidence

Comparison	Number of Studies	Within-study bias	Reporting bias	Indirectness	Imprecision	Heterogeneity	Incoherence	Confidence rating	Reason(s) for downgrading
**Aspirin vs Standard treatment**	--	No concerns<br>**Majority RoB:** No concerns<br>**Average RoB:** No concerns (applied)<br>**Highest RoB:** No concerns	Low risk<br>**Evidence:** mixed<br>**Source:** manual	No concerns	Major concerns<br>**NMA effect:** 0.259<br>**95% CI:** (0.046, 1.448)<br>crosses 2 clinically important line(s)<br>**Clinically important:** (0.8, 1.25)	No concerns<br>**NMA effect:** 0.259<br>**95% CI:** (0.046, 1.448)<br>**95% PrI:** (0.009, 7.409)	Major concerns<br>**Evidence:** indirect only<br>IF not applicable; judged from the design-by-treatment test.	Low	|
**Aspirin vs Ticagrelor**	--	No concerns<br>**Majority RoB:** No concerns<br>**Average RoB:** No concerns (applied)<br>**Highest RoB:** No concerns	Low risk<br>**Evidence:** mixed<br>**Source:** manual	No concerns	Major concerns<br>**NMA effect:** 0.614<br>**95% CI:** (0.089, 4.264)<br>crosses 2 clinically important line(s)<br>**Clinically important:** (0.8, 1.25)	No concerns<br>**NMA effect:** 0.614<br>**95% CI:** (0.089, 4.264)<br>**95% PrI:** (0.016, 23.660)	Major concerns<br>**Evidence:** indirect only<br>IF not applicable; judged from the design-by-treatment test.	Low	|

Comparison	Number of Studies	Within-study bias	Reporting bias	Indirectness	Imprecision	Heterogeneity	Incoherence	Confidence rating	Reason(s) for downgrading
**Clopidogrel vs Ticagrelor**	--	No concerns<br>**Majority RoB:** No concerns<br>**Average RoB:** No concerns (applied)<br>**Highest RoB:** No concerns	Low risk<br>**Evidence:** mixed<br>**Source:** manual	No concerns	Major concerns<br>**NMA effect:** 0.675<br>**95% CI:** (0.127, 3.590)<br>crosses 2 clinically important line(s)<br>**Clinically important:** (0.8, 1.25)	No concerns<br>**NMA effect:** 0.675<br>**95% CI:** (0.127, 3.590)<br>**95% PrI:** (0.025, 18.046)	Major concerns<br>**Evidence:** indirect only<br>IF not applicable; judged from the design-by-treatment test.	Low	
