# Supplementary material for: Antiplatelet monotherapy maintenance strategies after percutaneous coronary intervention in patients with coronary artery disease at high bleeding risk: a network meta-analysis
Source: Front Cardiovasc Med. 2026 Oct 28;13:1941814. doi: 10.3389/fcvm.2026.1941814 (PMC13751440; doi:10.3389/fcvm.2026.1941814)
Supplement: Supplementary file 4 [file Datasheet4.pdf]

Confidence In Network Meta Analysis - CINeMA 2.0.0 - Project: **Myocardial infarction**

Myocardial infarction

Comparison	Number of Studies	Within-study bias	Reporting bias	Indirectness	Imprecision	Heterogeneity	Incoherence	Confidence rating	Reason(s) for downgrading
Mixed evidence									
**Aspirin vs Clopidogrel**	2	No concerns<br>**Majority RoB:** No concerns<br>**Average RoB:** No concerns (applied)<br>**Highest RoB:** No concerns	Low risk<br>**Evidence:** mixed<br>**Source:** manual	No concerns<br>**Majority:** low<br>**Average:** low<br>**Highest:** low	Major concerns<br>**NMA effect:** 1.161<br>**95% CI:** (0.575, 2.343)<br>crosses 2 clinically important line(s)<br>**Clinically important:** (0.8, 1.25)	No concerns<br>**NMA effect:** 1.161<br>**95% CI:** (0.575, 2.343)<br>**95% PrI:** (0.371, 3.632)<br>**τ^2:** —<br>**I^2:** null%	No concerns<br>**Evidence:** direct only<br>IF not applicable; judged from the design-by-treatment test.	Low	|

Comparison	Number of Studies	Within-study bias	Reporting bias	Indirectness	Imprecision	Heterogeneity	Incoherence	Confidence rating	Reason(s) for downgrading
**Clopidogrel vs Standard treatment**	1	No concerns<br>**Majority RoB:** No concerns<br>**Average RoB:** No concerns (applied)<br>**Highest RoB:** No concerns	Low risk<br>**Evidence:** mixed<br>**Source:** manual	No concerns<br>**Majority:** low<br>**Average:** low<br>**Highest:** low	No concerns<br>**NMA effect:** 3.272<br>**95% CI:** (1.293, 8.279)<br>crosses 0 clinically important line(s)<br>**Clinically important:** (0.8, 1.25)	Major concerns<br>**NMA effect:** 3.272<br>**95% CI:** (1.293, 8.279)<br>**95% PrI:** (0.725, 14.773)<br>**τ²:** —<br>**I²:** null%	No concerns<br>**Evidence:** direct only<br>IF not applicable; judged from the design-by-treatment test.	Low	|
**Standard treatment vs Ticagrelor**	3	No concerns<br>**Majority RoB:** No concerns<br>**Average RoB:** No concerns (applied)<br>**Highest RoB:** No concerns	Low risk<br>**Evidence:** mixed<br>**Source:** manual	No concerns<br>**Majority:** low<br>**Average:** low<br>**Highest:** low	Major concerns<br>**NMA effect:** 0.969<br>**95% CI:** (0.643, 1.460)<br>crosses 2 clinically important line(s)<br>**Clinically important:** (0.8, 1.25)	No concerns<br>**NMA effect:** 0.969<br>**95% CI:** (0.643, 1.460)<br>**95% PrI:** (0.497, 1.885)<br>**τ²:** 0.000<br>**I²:** 0.0%%	No concerns<br>**Evidence:** direct only<br>IF not applicable; judged from the design-by-treatment test.	Low	|

Indirect evidence

Comparison	Number of Studies	Within-study bias	Reporting bias	Indirectness	Imprecision	Heterogeneity	Incoherence	Confidence rating	Reason(s) for downgrading
Aspirin vs Standard treatment	--	No concerns<br>**Majority RoB:** No concerns<br>**Average RoB:** No concerns (applied)<br>**Highest RoB:** No concerns	Low risk<br>**Evidence:** mixed<br>**Source:** manual	No concerns	No concerns<br>**NMA effect:** 3.797<br>**95% CI:** (1.185, 12.164)<br>crosses 0 clinically important line(s)<br>**Clinically important:** (0.8, 1.25)	Major concerns<br>**NMA effect:** 3.797<br>**95% CI:** (1.185, 12.164)<br>**95% PrI:** (0.573, 25.146)	No concerns<br>**Evidence:** indirect only<br>IF not applicable; judged from the design-by-treatment test.	Low	|
Aspirin vs Ticagrelor	--	No concerns<br>**Majority RoB:** No concerns<br>**Average RoB:** No concerns (applied)<br>**Highest RoB:** No concerns	Low risk<br>**Evidence:** mixed<br>**Source:** manual	No concerns	No concerns<br>**NMA effect:** 3.677<br>**95% CI:** (1.070, 12.637)<br>crosses 0 clinically important line(s)<br>**Clinically important:** (0.8, 1.25)	Major concerns<br>**NMA effect:** 3.677<br>**95% CI:** (1.070, 12.637)<br>**95% PrI:** (0.495, 27.292)	No concerns<br>**Evidence:** indirect only<br>IF not applicable; judged from the design-by-treatment test.	Low	|

Comparison	Number of Studies	Within-study bias	Reporting bias	Indirectness	Imprecision	Heterogeneity	Incoherence	Confidence rating	Reason(s) for downgrading
Clopidogrel vs Ticagrelor	--	No concerns<br>**Majority RoB:** No concerns<br>**Average RoB:** No concerns (applied)<br>**Highest RoB:** No concerns	Low risk<br>**Evidence:** mixed<br>**Source:** manual	No concerns	No concerns<br>**NMA effect:** 3.169<br>**95% CI:** (1.148, 8.743)<br>crosses 0 clinically important line(s)<br>**Clinically important:** (0.8, 1.25)	Major concerns<br>**NMA effect:** 3.169<br>**95% CI:** (1.148, 8.743)<br>**95% PrI:** (0.610, 16.468)	No concerns<br>**Evidence:** indirect only<br>IF not applicable; judged from the design-by-treatment test.	Low	
