# Supplementary material for: Antiplatelet monotherapy maintenance strategies after percutaneous coronary intervention in patients with coronary artery disease at high bleeding risk: a network meta-analysis
Source: Front Cardiovasc Med. 2026 Oct 28;13:1941814. doi: 10.3389/fcvm.2026.1941814 (PMC13751440; doi:10.3389/fcvm.2026.1941814)
Supplement: Supplementary file 5 [file Datasheet5.pdf]

# Confidence In Network Meta Analysis - CINeMA 2.0.0 - Project: **Stroke**

## Stroke

| Comparison | Number of Studies | Within-study bias | Reporting bias | Indirectness | Imprecision | Heterogeneity | Incoherence | Confidence rating | Reason(s) for downgrading |
|---|---|---|---|---|---|---|---|---|---|
| Mixed evidence | | | | | | | | | |
| **Aspirin vs Clopidogrel** | 2 | No concerns<br>**Majority RoB:** No concerns<br>**Average RoB:** No concerns (applied)<br>**Highest RoB:** No concerns | Low risk<br>**Evidence:** mixed<br>**Source:** manual | No concerns<br>**Majority:** low<br>**Average:** low<br>**Highest:** low | Major concerns<br>**NMA effect:** 1.348<br>**95% CI:** (0.453, 4.009)<br>crosses 2 clinically important line(s)<br>**Clinically important:** (0.8, 1.25) | No concerns<br>**NMA effect:** 1.348<br>**95% CI:** (0.453, 4.009)<br>**95% PrI:** (0.111, 16.336)<br>**$\tau^2$:** —<br>**$I^2$:** null% | No concerns<br>**Evidence:** direct only<br>IF not applicable; judged from the design-by-treatment test. | Low | |

| Comparison | Number of Studies | Within-study bias | Reporting bias | Indirectness | Imprecision | Heterogeneity | Incoherence | Confidence rating | Reason(s) for downgrading |
|---|---|---|---|---|---|---|---|---|---|
| **Clopidogrel vs Standard treatment** | 1 | No concerns<br>**Majority RoB:** No concerns<br>**Average RoB:** No concerns (applied)<br>**Highest RoB:** No concerns | Low risk<br>**Evidence:** mixed<br>**Source:** manual | No concerns<br>**Majority:** low<br>**Average:** low<br>**Highest:** low | Major concerns<br>**NMA effect:** 0.931<br>**95% CI:** (0.250, 3.473)<br>crosses 2 clinically important line(s)<br>**Clinically important:** (0.8, 1.25) | No concerns<br>**NMA effect:** 0.931<br>**95% CI:** (0.250, 3.473)<br>**95% PrI:** (0.059, 14.823)<br>**$\tau^2$:** —<br>**$I^2$:** null% | No concerns<br>**Evidence:** direct only<br>IF not applicable; judged from the design-by-treatment test. | Low | |
| **Standard treatment vs Ticagrelor** | 3 | No concerns<br>**Majority RoB:** No concerns<br>**Average RoB:** No concerns (applied)<br>**Highest RoB:** No concerns | Low risk<br>**Evidence:** mixed<br>**Source:** manual | No concerns<br>**Majority:** low<br>**Average:** low<br>**Highest:** low | Major concerns<br>**NMA effect:** 1.007<br>**95% CI:** (0.315, 3.222)<br>crosses 2 clinically important line(s)<br>**Clinically important:** (0.8, 1.25) | No concerns<br>**NMA effect:** 1.007<br>**95% CI:** (0.315, 3.222)<br>**95% PrI:** (0.076, 13.290)<br>**$\tau^2$:** 0.000<br>**$I^2$:** 0.0%% | No concerns<br>**Evidence:** direct only<br>IF not applicable; judged from the design-by-treatment test. | Low | |

Indirect evidence

| Comparison | Number of Studies | Within-study bias | Reporting bias | Indirectness | Imprecision | Heterogeneity | Incoherence | Confidence rating | Reason(s) for downgrading |
|---|---|---|---|---|---|---|---|---|---|
| Aspirin vs Standard treatment | -- | No concerns<br>**Majority RoB:** No concerns<br>**Average RoB:** No concerns (applied)<br>**Highest RoB:** No concerns | Low risk<br>**Evidence:** mixed<br>**Source:** manual | No concerns | Major concerns<br>**NMA effect:** 1.255<br>**95% CI:** (0.227, 6.932)<br>crosses 2 clinically important line(s)<br>**Clinically important:** (0.8, 1.25) | No concerns<br>**NMA effect:** 1.255<br>**95% CI:** (0.227, 6.932)<br>**95% PrI:** (0.047, 33.525) | No concerns<br>**Evidence:** indirect only<br>IF not applicable; judged from the design-by-treatment test. | Low | |
| Aspirin vs Ticagrelor | -- | No concerns<br>**Majority RoB:** No concerns<br>**Average RoB:** No concerns (applied)<br>**Highest RoB:** No concerns | Low risk<br>**Evidence:** mixed<br>**Source:** manual | No concerns | Major concerns<br>**NMA effect:** 1.264<br>**95% CI:** (0.160, 9.986)<br>crosses 2 clinically important line(s)<br>**Clinically important:** (0.8, 1.25) | No concerns<br>**NMA effect:** 1.264<br>**95% CI:** (0.160, 9.986)<br>**95% PrI:** (0.029, 55.880) | No concerns<br>**Evidence:** indirect only<br>IF not applicable; judged from the design-by-treatment test. | Low | |

| Comparison | Number of Studies | Within-study bias | Reporting bias | Indirectness | Imprecision | Heterogeneity | Incoherence | Confidence rating | Reason(s) for downgrading |
|---|---|---|---|---|---|---|---|---|---|
| **Clopidogrel vs Ticagrelor** | -- | No concerns<br>**Majority RoB:** No concerns<br>**Average RoB:** No concerns (applied)<br>**Highest RoB:** No concerns | Low risk<br>**Evidence:** mixed<br>**Source:** manual | No concerns | Major concerns<br>**NMA effect:** 0.938<br>**95% CI:** (0.162, 5.430)<br>crosses 2 clinically important line(s)<br>**Clinically important:** (0.8, 1.25) | No concerns<br>**NMA effect:** 0.938<br>**95% CI:** (0.162, 5.430)<br>**95% PrI:** (0.033, 26.733) | No concerns<br>**Evidence:** indirect only<br>IF not applicable; judged from the design-by-treatment test. | Low | |
