# Supplementary material for: Antiplatelet monotherapy maintenance strategies after percutaneous coronary intervention in patients with coronary artery disease at high bleeding risk: a network meta-analysis
Source: Front Cardiovasc Med. 2026 Oct 28;13:1941814. doi: 10.3389/fcvm.2026.1941814 (PMC13751440; doi:10.3389/fcvm.2026.1941814)
Supplement: Supplementary file 6 [file Datasheet6.pdf]

PUBMED

#1:"CAD"[Title/Abstract] OR "CHD"[Title/Abstract] OR "Coronary Arterioscleroses"[Title/Abstract] OR "Coronary Arteriosclerosis"[Title/Abstract] OR "Coronary Artery Disease"[MeSH Terms] OR "Coronary Artery Disease"[Title/Abstract] OR "Coronary Artery Diseases"[Title/Abstract] OR "Coronary Atheroscleroses"[Title/Abstract] OR "Coronary Atherosclerosis"[Title/Abstract] OR "Left Main Coronary Artery Disease"[Title/Abstract] OR "Left Main Coronary Disease"[Title/Abstract] OR "Left Main Disease"[Title/Abstract] OR "Left Main Diseases"[Title/Abstract] OR "PCI"[Title/Abstract] OR "PTCA"[Title/Abstract] OR "Percutaneous Coronary Intervention"[MeSH Terms] OR "Percutaneous Coronary Intervention"[Title/Abstract] OR "Percutaneous Coronary Interventions"[Title/Abstract] OR "Percutaneous Coronary Revascularization"[Title/Abstract] OR "Percutaneous Coronary Revascularizations"[Title/Abstract] OR "angioplasty"[Title/Abstract] OR "ischemic heart disease"[Title/Abstract]
#2:"Blood Platelet Aggregation Inhibitor"[Title/Abstract] OR "Blood Platelet Aggregation Inhibitors"[Title/Abstract] OR "Blood Platelet Antagonist"[Title/Abstract] OR "Blood Platelet Antagonists"[Title/Abstract] OR "Blood Platelet Antiaggregant"[Title/Abstract] OR "Blood Platelet Antiaggregants"[Title/Abstract] OR "GP IIb/IIIa inhibitor"[Title/Abstract] OR "P2Y12 inhibitor"[Title/Abstract] OR "PAR 1 Antagonists"[Title/Abstract] OR "Platelet Aggregation Inhibitor"[Title/Abstract] OR "Platelet Aggregation Inhibitors"[MeSH Terms] OR "Platelet Antagonist"[Title/Abstract] OR "Platelet Antagonists"[Title/Abstract] OR "Platelet Antiaggregant"[Title/Abstract] OR "Platelet Antiaggregants"[Title/Abstract] OR "Platelet Inhibitor"[Title/Abstract] OR "Platelet Inhibitors"[Title/Abstract] OR "antiplatelet"[Title/Abstract]
#3:"Adverse Cardiac Event"[Title/Abstract] OR "Adverse Cardiac Events"[Title/Abstract] OR "BARC"[Title/Abstract] OR "Bleeding"[Title/Abstract] OR "Bloodletting"[Title/Abstract] OR "CV death"[Title/Abstract] OR "Cardiac Event"[Title/Abstract] OR "Cardiac Events"[Title/Abstract] OR "Cardiovascular Disease"[Title/Abstract] OR "Cardiovascular Diseases"[MeSH Terms] OR "Hemorrhage"[MeSH Terms] OR "Hemorrhages"[Title/Abstract] OR "MACCE"[Title/Abstract] OR "MACE"[Title/Abstract] OR "Major Adverse Cardiac Events"[Title/Abstract] OR "Phlebotomy"[Title/Abstract] OR "TIMI"[Title/Abstract] OR "major adverse cardiovascular event"[Title/Abstract]
#4:("RCT"[Title/Abstract] OR "Randomized Controlled Trial"[Publication Type] OR "clinical trial"[Publication Type] OR "randomised controlled trial"[Title/Abstract]) OR ("Clinical Trial"[Publication Type] OR "Randomized Controlled Trial"[Publication Type])
#5:#1 AND #2 AND #3 AND #4

Embase

#1:('CAD':ti,ab OR 'CHD':ti,ab OR 'Coronary Arterioscleroses':ti,ab OR 'Coronary Arteriosclerosis':ti,ab OR 'Coronary Artery Disease':ti,ab OR 'Coronary Artery Diseases':ti,ab OR 'Coronary Atheroscleroses':ti,ab OR 'Coronary Atherosclerosis':ti,ab OR 'Left Main Coronary Artery Disease':ti,ab OR 'Left Main Coronary Disease':ti,ab OR 'Left Main Disease':ti,ab OR 'Left Main Diseases':ti,ab OR 'PCI':ti,ab OR 'PTCA':ti,ab OR 'Percutaneous Coronary Intervention':ti,ab OR 'Percutaneous Coronary Interventions':ti,ab OR 'Percutaneous Coronary Revascularization':ti,ab OR 'Percutaneous Coronary Revascularizations':ti,ab OR 'angioplasty':ti,ab OR 'ischemic heart disease':ti,ab) OR ('Coronary Artery Disease'/exp OR 'Percutaneous Coronary Intervention'/exp)
#2:('Blood Platelet Aggregation Inhibitor':ti,ab OR 'Blood Platelet Aggregation Inhibitors':ti,ab OR 'Blood Platelet Antagonist':ti,ab OR 'Blood Platelet Antagonists':ti,ab OR 'Blood Platelet Antiaggregant':ti,ab OR 'Blood Platelet Antiaggregants':ti,ab OR 'GP IIb/IIIa inhibitor':ti,ab OR 'P2Y12 inhibitor':ti,ab OR 'PAR 1 Antagonists':ti,ab OR 'Platelet Aggregation Inhibitor':ti,ab OR 'Platelet Antagonist':ti,ab OR 'Platelet Antagonists':ti,ab OR 'Platelet Antiaggregant':ti,ab OR 'Platelet Antiaggregants':ti,ab OR 'Platelet Inhibitor':ti,ab OR 'Platelet Inhibitors':ti,ab OR 'antiplatelet':ti,ab) OR ('Platelet Aggregation Inhibitors'/exp)
#3:('Adverse Cardiac Event':ti,ab OR 'Adverse Cardiac Events':ti,ab OR 'BARC':ti,ab OR 'Bleeding':ti,ab OR 'Bloodletting':ti,ab OR 'CV death':ti,ab OR 'Cardiac Event':ti,ab OR 'Cardiac Events':ti,ab OR 'Cardiovascular Disease':ti,ab OR 'Hemorrhages':ti,ab OR 'MACCE':ti,ab OR 'MACE':ti,ab OR 'Major Adverse Cardiac Events':ti,ab OR 'Phlebotomy':ti,ab OR 'TIMI':ti,ab OR 'major adverse cardiovascular event':ti,ab) OR ('Cardiovascular Diseases'/exp OR 'Hemorrhage'/exp)
#4:('RCT':ti,ab OR 'randomised controlled trial':ti,ab) OR ('Randomized Controlled Trial':it OR 'clinical trial':it)
#5:#1 AND #2 AND #3 AND #4

Web of Science

TS=("CAD" OR "CHD" OR "Coronary Arterioscleroses" OR "Coronary Arteriosclerosis" OR "Coronary Artery Disease" OR "Coronary Artery Diseases" OR "Coronary Atheroscleroses" OR "Coronary Atherosclerosis" OR "Left Main Coronary Artery Disease" OR "Left Main Coronary Disease" OR "Left Main Disease" OR "Left Main Diseases" OR "PCI" OR "PTCA" OR "Percutaneous Coronary Intervention" OR "Percutaneous Coronary Interventions" OR "Percutaneous Coronary Revascularization" OR "Percutaneous Coronary Revascularizations" OR "angioplasty" OR "ischemic heart disease") AND TS=("Blood Platelet Aggregation Inhibitor" OR "Blood Platelet Aggregation Inhibitors" OR "Blood Platelet Antagonist" OR "Blood Platelet Antagonists" OR "Blood Platelet Antiaggregant" OR "Blood Platelet Antiaggregants" OR "GP IIb/IIIa inhibitor" OR "P2Y12 inhibitor" OR "PAR 1 Antagonists" OR "Platelet Aggregation Inhibitor" OR "Platelet Aggregation Inhibitors"

OR "Platelet Antagonist" OR "Platelet Antagonists" OR "Platelet Antiaggregant" OR "Platelet Antiaggregants" OR "Platelet Inhibitor" OR "Platelet Inhibitors" OR "antiplatelet") AND TS=("Adverse Cardiac Event" OR "Adverse Cardiac Events" OR "BARC" OR "Bleeding" OR "Bloodletting" OR "CV death" OR "Cardiac Event" OR "Cardiac Events" OR "Cardiovascular Disease" OR "Cardiovascular Diseases" OR "Hemorrhage" OR "Hemorrhages" OR "MACCE" OR "MACE" OR "Major Adverse Cardiac Events" OR "Phlebotomy" OR "TIMI" OR "major adverse cardiovascular event") AND (TS=("RCT") OR DT=("Randomized Controlled Trial") OR DT=("clinical trial") OR TS=("randomised controlled trial"))

Cochrane Central

#1:("CAD" OR "CHD" OR "Coronary Arterioscleroses" OR "Coronary Arteriosclerosis" OR "Coronary Artery Disease" OR "Coronary Artery Diseases" OR "Coronary Atheroscleroses" OR "Coronary Atherosclerosis" OR "Left Main Coronary Artery Disease" OR "Left Main Coronary Disease" OR "Left Main Disease" OR "Left Main Diseases" OR "PCI" OR "PTCA" OR "Percutaneous Coronary Intervention" OR "Percutaneous Coronary Interventions" OR "Percutaneous Coronary Revascularization" OR "Percutaneous Coronary Revascularizations" OR "angioplasty" OR "ischemic heart disease"):ti,ab,kw
#2: [mh "Coronary Artery Disease"]
#3: [mh "Percutaneous Coronary Intervention"]
#4: ("Blood Platelet Aggregation Inhibitor" OR "Blood Platelet Aggregation Inhibitors" OR "Blood Platelet Antagonist" OR "Blood Platelet Antagonists" OR "Blood Platelet Antiaggregant" OR "Blood Platelet Antiaggregants" OR "GP IIb/IIIa inhibitor" OR "P2Y12 inhibitor" OR "PAR 1 Antagonists" OR "Platelet Aggregation Inhibitor" OR "Platelet Antagonist" OR "Platelet Antagonists" OR "Platelet Antiaggregant" OR "Platelet Antiaggregants" OR "Platelet Inhibitor" OR "Platelet Inhibitors" OR "antiplatelet"):ti,ab,kw
#5: [mh "Platelet Aggregation Inhibitors"]
#6: ("Adverse Cardiac Event" OR "Adverse Cardiac Events" OR "BARC" OR "Bleeding" OR "Bloodletting" OR "CV death" OR "Cardiac Event" OR "Cardiac Events" OR "Cardiovascular Disease" OR "Hemorrhages" OR "MACCE" OR "MACE" OR "Major Adverse Cardiac Events" OR "Phlebotomy" OR "TIMI" OR "major adverse cardiovascular event"):ti,ab,kw
#7: [mh "Cardiovascular Diseases"]
#8: [mh Hemorrhage]
#9: ("RCT" OR "randomised controlled trial"):ti,ab,kw
#10: ("Randomized Controlled Trial" OR "clinical trial"):pt
#11: (#1 OR #2 OR #3) AND (#4 OR #5) AND (#6 OR #7 OR #8) AND (#9 OR #10)
